# Oral contraceptive use and risk of liver cancer: a population-based study, systematic review, and meta-analysis

**DOI:** 10.1016/S1470-2045(25)00222-0

**Published:** 2025-08

**Authors:** Cody Z Watling, Siân Sweetland, Aika Wojt, Gisela Butera, Barry I Graubard, Sarah Floud, Charles E Matthews, Gillian K Reeves, Katherine A McGlynn

**Affiliations:** a Metabolic Epidemiology Branch, Division of Cancer Epidemiology and Genetics, National Cancer Institute, Rochville, MD, USA; b Biostatistics Branch, Division of Cancer Epidemiology and Genetics, National Cancer Institute, Rochville, MD, USA; c Cancer Epidemiology Unit, Nuffield Department of Population Health, University of Oxford, Oxford, UK; d Office of Research Services, Division of Library Services, National Institutes of Health Library, Bethesda, MD, USA

## Abstract

**Background:**

Oral contraceptive use has been suggested to increase the risk of liver cancer. Although the International Agency for Research on Cancer concluded in 1999 that there was sufficient evidence of an association, this was based on case–control studies with few liver cancer cases. We aimed to provide more robust epidemiological evidence on this association by analysing data from two large prospective UK cohorts and additionally conducting a systematic review and meta-analysis of previous observational studies.

**Methods:**

In our population-based study, the relationship between oral contraceptive use and liver cancer risk was examined using data from the Million Women Study (MWS) and the UK Biobank. We included women from both cohorts who did not have a prevalent cancer at baseline (except non-melanoma skin cancer) and had provided data on oral contraceptive use; incident liver cancer diagnoses were determined using linkage to National Health Service cancer registries. We compared risk in women who had ever used oral contraceptives with women who had never used oral contraceptives. Multivariable Cox proportional hazards regression was used to calculate hazard ratios (HRs) and 95% CIs. For the systematic review and meta-analysis, we searched PubMed, Embase, CINAHL Plus, Web of Science, and Scopus from database inception to June 28, 2024, for existing observational studies. Study-specific log odds ratios (ORs) or log HRs were pooled and we determined the relative risk (RR) between oral contraceptive use and liver cancer across all studies using a fixed-effects model (PROSPERO number CRD42024552518).

**Findings:**

A total of 2765 (0·21%) of 1 305 024 participants developed liver cancer in the MWS cohort (median follow-up 21·4 years; IQR 18·4–22·4) and 191 (0·08%) of 253 408 participants developed liver cancer in the UK Biobank (median follow-up 12·6 years; IQR 11·8–13·4). No association was observed between ever versus never use of oral contraceptives and liver cancer risk in either the MWS (HR 1·05, 95% CI 0·97–1·13; p=0·27) or the UK Biobank (1·08, 0·76–1·55; p=0·66). The meta-analysis of 23 observational studies, which included 5422 individuals with liver cancer, found no evidence of an association between ever versus never oral contraceptive use and liver cancer (RR 1·04, 0·98–1·11; *I*^2^ 45·9%, p=0·0080). In the meta-analysis of duration of oral contraceptive use, there was a slightly increased risk of liver cancer per 5 years of use of oral contraceptives (RR 1·06, 1·02–1·10; *I*^2^ 63·9%, p<0·0001), with corresponding subtype-specific RRs of 1·07 (1·00–1·14) for hepatocellular carcinoma and 1·06 (1·01–1·11) for intrahepatic cholangiocarcinoma (p_heterogeneity_=0·82).

**Interpretation:**

The totality of observational studies suggests there is no association between ever versus never use of oral contraceptive and liver cancer risk. When looking at associations by duration of oral contraceptive use, there was little or no association with all liver cancer or its two main subtypes. There might be a small increased risk of liver cancer with longer duration of use, but residual confounding cannot be ruled out.

**Funding:**

Canadian Institutes of Health Research, National Institutes of Health Intramural Program, and Cancer Research UK.

## Introduction

Liver cancer is the third leading cause of cancer-related deaths globally,^1^ and the number of cases worldwide is projected to increase by approximately 55% by 2040.^2^ Hepatocellular carcinoma is the predominant histological type of liver cancer in most regions of the world, constituting 75–85% of all cases, whereas intrahepatic cholangiocarcinoma is the second most common histological type, representing 10–15% of cases.^2^ In most regions, the incidence of primary liver cancer is 2 to 3 times lower in female individuals than in male individuals. The sex difference in liver cancer rates is primarily due to the difference in hepatocellular carcinoma rates, as intrahepatic cholangiocarcinoma rates in male and female individuals are much more similar.^2^, ^3^ Although major risk factors for liver cancer, such as chronic hepatitis B virus (HBV) and hepatitis C virus (HCV) infection, excessive alcohol intake, and cigarette smoking, are more common in male individuals, these factors do not completely explain the sex-based difference in incidence.^4^ The suggestion that hormonal factors might influence liver cancer risk has therefore been proposed to further explain this difference.

**Research in context**

**Evidence before this study**

In 1999, the International Agency for Research on Cancer (IARC) first stated that there was sufficient evidence that oral contraceptive pills cause hepatocellular carcinoma, the main subtype of liver cancer, in the absence of viral hepatitis infections. This statement was further reinstated in a 2012 IARC report on pharmaceuticals and cancer risk, which stated that long-term use of oral contraceptives was associated with hepatocellular carcinoma risk. However, the evidence from human studies was primarily from small case–control studies with few participants with liver cancer. As liver cancer is relatively rare in female individuals, as compared with both other cancers and male individuals (∼6 cases per 100 000 individuals per year in the UK), few prospective studies have had a sufficient number of participants to assess the association between oral contraceptive use and liver cancer risk. In this study, we analysed data from two of the largest UK prospective cohorts, the Million Women Study (MWS) and the UK Biobank, which combined have nearly 3000 participants with liver cancer, to assess the association between oral contraceptive use and liver cancer risk. We then assessed all existing observational evidence on the association between oral contraceptive use and liver cancer risk to date in a systematic review and meta-analysis.

**Added value of this study**

Our study found no evidence of an association between oral contraceptive use and risk of liver cancer in the MWS or in the UK Biobank. Our systematic review and meta-analysis of previous observational studies plus our findings from the MWS and the UK Biobank showed no difference in associated liver cancer risk between women who had ever used oral contraceptives and women who had never used them. When we explored studies that assessed oral contraceptive use by duration, women who used oral contraceptives for longer were found to be at a slightly increased risk of liver cancer.

**Implications of all available evidence**

This study suggests that there is little to no evidence to support an association between oral contraceptive use and risk of liver cancer. There might be a small positive association between longer oral contraceptive use and liver cancer risk, however, this could be due to other reasons, such as inadequate adjustment for confounders.

In the 1970s, numerous case reports were published linking the use of oral contraceptives to benign liver tumours in women (ie, hepatocellular adenomas and focal nodular hyperplasia).^5^, ^6^ As benign liver tumours can undergo malignant transformation, these findings led to the hypothesis that oral contraceptive use could also be a risk factor for malignant liver cancer. In 1999, an expert review panel of the International Agency for Research on Cancer (IARC) concluded that there was sufficient evidence that oral contraceptive use increased the risk of liver cancer in the absence of viral exposures (ie, HBV and HCV).^7^ Updates by IARC expert review panels in 2007 and 2012, supported the original conclusion of sufficient evidence of an association.^8^, ^9^ The evidence supporting the conclusions was largely based on case–control studies as few cases were yet to accrue in the then existing prospective studies. However, case–control studies are subject to recall bias of reported exposures and risk factors or covariates. Since 2012, sufficient liver cancer outcomes have accrued in prospective studies to assess associations with oral contraceptive usage.

The aim of this study is to provide more robust epidemiological evidence on the association of oral contraceptives and liver cancer risk. To this end, we analysed data from two of the largest prospective UK cohorts, the Million Women Study (MWS) and the UK Biobank, which together included over 1·5 million women. In addition, we conducted a systematic review and meta-analysis of research findings from previously published observational studies plus the findings from the MWS and UK Biobank cohorts.

## Methods

### Study design

Both the MWS and the UK Biobank are prospective cohorts. The MWS includes around 1·3 million women (aged 50–64 years) recruited in the UK with over 20 years of follow-up. Further details regarding the MWS cohort have been described elsewhere.^10^ Briefly, between 1996 and 2001, over 1·3 million women attending the National Health Service (NHS) Breast Screening Programme across 66 centres consented to take part in the MWS. Women were sent a recruitment questionnaire that asked about sociodemographic, lifestyle, and health factors, and have been resurveyed every 3–5 years since recruitment. Ethical approval was provided by the East of England-Cambridge South Research Ethics Committee (REC 97/5/001).

The UK Biobank is a prospective cohort of around half a million UK adults who have been followed up for 12 years. Briefly, from 2006 to 2010, 9·2 million individuals from across the UK were contacted via NHS patient registers, of which 503 317 individuals aged 37–73 years consented to enrol in the study. Approximately half of the participants (∼270 000) are women. At recruitment, participants attended an assessment centre, provided informed consent, and answered questions about their lifestyle, sociodemographic, and reproductive factors via a touchscreen questionnaire; in addition, anthropometric measurements were taken using standardised procedures. Further details regarding the UK Biobank have been described elsewhere.^11^, ^12^ The UK Biobank has received ethical approval from the Northwest Multi-Centre Research Ethics Committee (reference number 21/NW/0157).

### Participants

All participants who were recruited to the MWS with consent to follow-up and all female UK Biobank participants were eligible to be included in this study. For the MWS, we excluded women who had a prevalent cancer at baseline other than non-melanoma skin cancer and who did not answer questions about oral contraceptive use. For UK Biobank, we excluded participants with a prevalent cancer at baseline (except non-melanoma skin cancer), male individuals, or women with missing information on oral contraceptive use. The full exclusion criteria applied for the MWS and UK Biobank are available in the appendix (pp 25–26).

Questions on oral contraceptive use were included in the baseline survey in the MWS and UK Biobank. Participants were asked if they ever used oral contraceptives, and for women who answered “yes”, questions were asked on duration of use, age at first use, and age at last use.

Participants were followed up through linkage to national medical records and all participants provided consent at recruitment to the studies. No additional consent was required for this analysis.

### Procedures

Incident liver cancer diagnoses were determined using linkage to NHS cancer registries. Specifically, data were provided from NHS England for participants in England and Wales, and the NHS Central Register for participants in Scotland. Participants contributed follow-up time from date of recruitment until the date of first registration of cancer (excluding non-melanoma skin cancer [ICD-10 code C44]), date of death, or last day of follow-up from the cancer registry, whichever came first. The MWS date for end of follow-up was Dec 31, 2020. The UK Biobank dates for end of follow-up were Dec 31, 2020, for participants in England; Nov 30, 2021, for participants in Scotland; and Dec 31, 2016, for participants in Wales. Participants were coded as having a primary event if they had an incident diagnosis of liver cancer (ICD-10 code C22). We only included incident liver cancer cases from the cancer registry and not from death records with no previous record due to potential coding issues (eg, death due to metastatic spread from another primary cancer site coded as death due to liver cancer). If available, liver cancer histological subtypes were determined using ICD-O-3 morphology codes—ie, for hepatocellular carcinoma, codes 8170–8175, and for intrahepatic cholangiocarcinoma, codes 8032–8033, 8041, 8050, 8070–8071, 8140–8141, 8160, 8260, 8480, 8481, and 8490.

### Statistical analyses

Baseline characteristics of women who never used oral contraceptives versus women who had ever used oral contraceptives were compared. Cox proportional hazards regression models with time on study as the underlying time variable were used to estimate hazard ratios (HRs) and 95% CIs for oral contraceptive use and liver cancer. We compared risk in women who had ever used oral contraceptives with women who had never used oral contraceptives. We also compared risk according to categories of duration of oral contraceptive use, defined as never used, less than 5 years of use, 5–9 years of use, or 10 or more years of use.

Covariates were a priori selected based on established risk factors for liver cancer (eg, alcohol intake, smoking, and diabetes), which have previously been associated with liver cancer in these cohorts,^13^, ^14^, ^15^ or theoretically important confounders between the association of oral contraceptive use and liver cancer risk (eg, previous hysterectomy). In MWS, minimally adjusted models were stratified by year of recruitment (ie, within period 1996–2001) and year of birth (≤1932, single years 1933–1949, and ≥1950); and adjusted for material deprivation (Townsend deprivation index quintiles) and region of recruitment (based on ten broad regions from the cancer registry). Minimally adjusted Cox regression models in the UK Biobank were adjusted by region of recruitment (North-West England, North-East England, Yorkshire and the Humber, West Midlands, East Midlands, South-East England, South-West England, London, Wales, and Scotland) and deprivation (Townsend deprivation index quintiles from most deprived to least deprived) and stratified by age at recruitment (<45 years, 45–49 years, 50–54 years, 55–59 years, 60–64 years, and ≥65 years).

Multivariable Cox regression models were further adjusted for BMI, height, physical activity, smoking status, alcohol consumption, education, diabetes status (type not specified), menopausal hormone therapy use, and hysterectomy status. For MWS, models were also adjusted for menopausal status and years since menopause for postmenopausal women, whereas for UK Biobank, models were adjusted for menopausal status (premenopausal, postmenopausal [with time since menopause], or unknown) as well as coffee intake and ethnicity. In the MWS, diabetes status was based on self-reported diagnosis of diabetes or treatment for diabetes; whereas in UK Biobank, diabetes status was based on self-reported diagnosis of diabetes, recorded use of diabetes-related medications, and measured HbA_1c_ at recruitment. Details regarding covariate classification and categorisation for MWS and UK Biobank can be found in the appendix (pp 2–8). A test for trend in risk with duration of oral contraceptive use was determined by assigning the median duration within each category (ie, <5 years, 5–9 years, ≥10 years) to all women within each category and modelling this as a linear variable.

In addition to total liver cancer, we also assessed associations by liver cancer histology, namely hepatocellular carcinoma and intrahepatic cholangiocarcinoma, and compared HRs and 95% CIs. We also assessed whether associations differed by age groups (ie, ≤55 years and >55 years) and by diabetes status. Formal tests for heterogeneity were conducted using a χ^2^ test comparing the HRs for oral contraceptive use (ever use *vs* never use) and risk of liver cancer between the subgroups of interest. In sensitivity analyses, we excluded the first 4 years of follow-up to assess whether the associations were influenced by latent disease.

### Search strategy and selection criteria

For the systematic review and meta-analysis, we searched PubMed, Embase, CINAHL Plus, Web of Science, and Scopus from database inception to June 28, 2024, with results restricted to English language publications. Key search terms included “exogenous hormone use” and “oral contraceptives”, as well as “liver cancer”, “hepatocellular carcinoma”, and “intrahepatic cholangiocarcinoma”. Both case–control and cohort study designs were included. Trial registries were not searched but we did search for unpublished data, none of which were included. Further details regarding the systematic review and meta-analysis of observational studies, including the full search strategy, flow diagram of results, and risk of bias are provided in the appendix (pp 8–15). One author conducted the library search to retrieve articles (GB) and two authors reviewed the articles and did the data extraction (CZW and AW). If there were any conflicts, the two reviewers tried to come to a consensus, but if the conflict was still unresolved a third reviewer (KAM) had the final say. Articles were included if they assessed associations between oral contraceptive use and liver cancer risk (or subtypes of liver cancer); were presented in English; and reported HRs, odds ratios (ORs), or relative risk (RR), with 95% CIs, or presented the number of cases and controls within each category, from which we could estimate ORs and 95% CIs. The systematic review was conducted per the PRISMA reporting checklist, which is available in the appendix (pp 36–38).^16^

### Data analysis

Study-specific log ORs or log HRs were pooled for use of oral contraceptives and a summarised effect size, referred to as RR, for oral contraceptive use was determined using a fixed-effects model. We also meta-analysed studies based on study design (ie, case–control *vs* prospective cohort) and by liver cancer subtype, hepatocellular carcinoma or intrahepatic cholangiocarcinoma (if presented), to see if associations differed. Studies that reported associations by duration of oral contraceptive use were also meta-analysed and we assessed the associations separately for hepatocellular carcinoma or intrahepatic cholangiocarcinoma. We calculated study-specific incremental estimates per 5 years of oral contraceptive use using generalised least squares,^17^ with the exception of MWS and UK Biobank, which were estimated from multivariable models per 5 years of use. Study-specific log RRs per 5 years of oral contraceptive use were pooled to obtain a summarised effect size for oral contraceptive use duration via a fixed-effects model. We also conducted a meta-analysis comparing the highest category of duration of use with never use of oral contraceptives. The *I*^2^ statistic was used to assess between-study heterogeneity for case–control studies, cohort studies, as well as all studies, and publication bias was also assessed using funnel plots and Egger's test.

All analyses were conducted using Stata 18·0 or StataNOW. All tests of significance were two-sided and p values <0·05, without adjustment for multiple comparisons, were considered statistically significant. The systematic review was prospectively registered in the PROSPERO database on June 22, 2024, (CRD42024552518; appendix pp 33–35).

### Role of the funding source

The funder of the study had no role in study design, data collection, data analysis, data interpretation, or writing of the report.

## Results

The MWS cohort had 1 364 256 women, we excluded women who had a prevalent cancer at baseline other than non-melanoma skin cancer (n=44 431) or did not answer questions about oral contraceptive use (n=14 801; appendix p 25). The UK Biobank cohort had 503 317 participants, 961 participants withdrew consent or were ineligible and we excluded participants with a prevalent cancer at baseline (except non-melanoma skin cancer; n=28 294), male individuals (n=219 303), or women with missing information on oral contraceptive use (n=1351; appendix p 26). After exclusions, a total of 1 305 024 from the MWS and 253 408 women from the UK Biobank were included.

After a median of 21·4 years (IQR 18·4–22·4) of follow-up, 2765 (0·21%) of 1 305 024 participants had incident liver cancer in the MWS. After a median of 12·6 years (11·8–13·4) of follow-up, 191 (0·08%) of 253 408 participants had incident liver cancer in the UK Biobank. Baseline characteristics of participants who had ever used oral contraceptives versus those who had never used oral contraceptives are presented in the table. In comparison with participants who had never used oral contraceptives, women who had ever used oral contraceptives in both MWS and UK Biobank were, on average, younger, more likely to consume more alcohol, more likely to be current smokers, less likely to have diabetes, and more likely to use menopausal hormone therapy.

**Table:** Baseline characteristics of participants in the Million Women Study and UK Biobank according to oral contraceptive use

	**Million Women Study**		**UK Biobank**	
	Never use of oral contraceptives (n=530 974)	Ever use of oral contraceptives (n=774 050)	Never use of oral contraceptives (n=47 290)	Ever use of oral contraceptives (n=206 118)
Age (years)	58·0 (4·9)	54·9 (4·4)	59·9 (7·9)	55·3 (7·8)
BMI (kg/m^2^)	26·5 (4·8)	26·1 (4·6)	27·5 (5·4)	27·0 (5·2)
Height (cm)	161·9 (6·7)	162·1 (6·6)	161·3 (6·4)	162·7 (6·3)
Alcoholic drinks per week (for those who drink alcohol)	5·6 (5·0)	6·6 (5·7)	12·1 (10·4)	14·1 (11·5)
No coffee intake	..	..	11 715 (24·8%)	48 497 (23.5%)
Currently smokes	89 122 (16·8%)	162 337 (21·0%)	3 189 (6·7%)	19 454 (9·4%)
Has diabetes (type not specified)	18 696 (3·5%)	17 111 (2·2%)	6380 (13·5%)	21 744 (10·5%)
Least deprived quintile	100 655 (19·0%)	161 102 (20·8%)	8 741 (18·5%)	41 836 (20·3%)
Highest physical activity category	31 181 (5·9%)	47 562 (6·1%)	8 415 (17·8%)	35 346 (17·1%)
Age at menarche (years)	13·0 (1·6)	13·0 (1·6)	13·0 (1·7)	13·0 (1·6)
Age at menopause (years)	48·0 (5·6)	47·7 (5·2)	46·1 (14·0)	46·6 (13·2)
Postmenopausal*	441 195/484 692 (91·0%)	496 336/616 852 (80·5%)	39 379 (83·3%)	140 018 (67·9%)
Current menopausal hormone therapy use*	110 257/436 732 (25·2%)	177 300/491 404 (36·1%)	1 816 (3·8%)	13 803 (6·7%)
Had a hysterectomy	134 522 (25·3%)	188 431 (24·3%)	4 451 (9·4%)	12 364 (6·0%)

Data are n (%) or mean (SD).

* Percentages for postmenopausal and menopausal hormone therapy use excluded participants with unknown or missing information in the Million Women Study.

**Figure 1:**
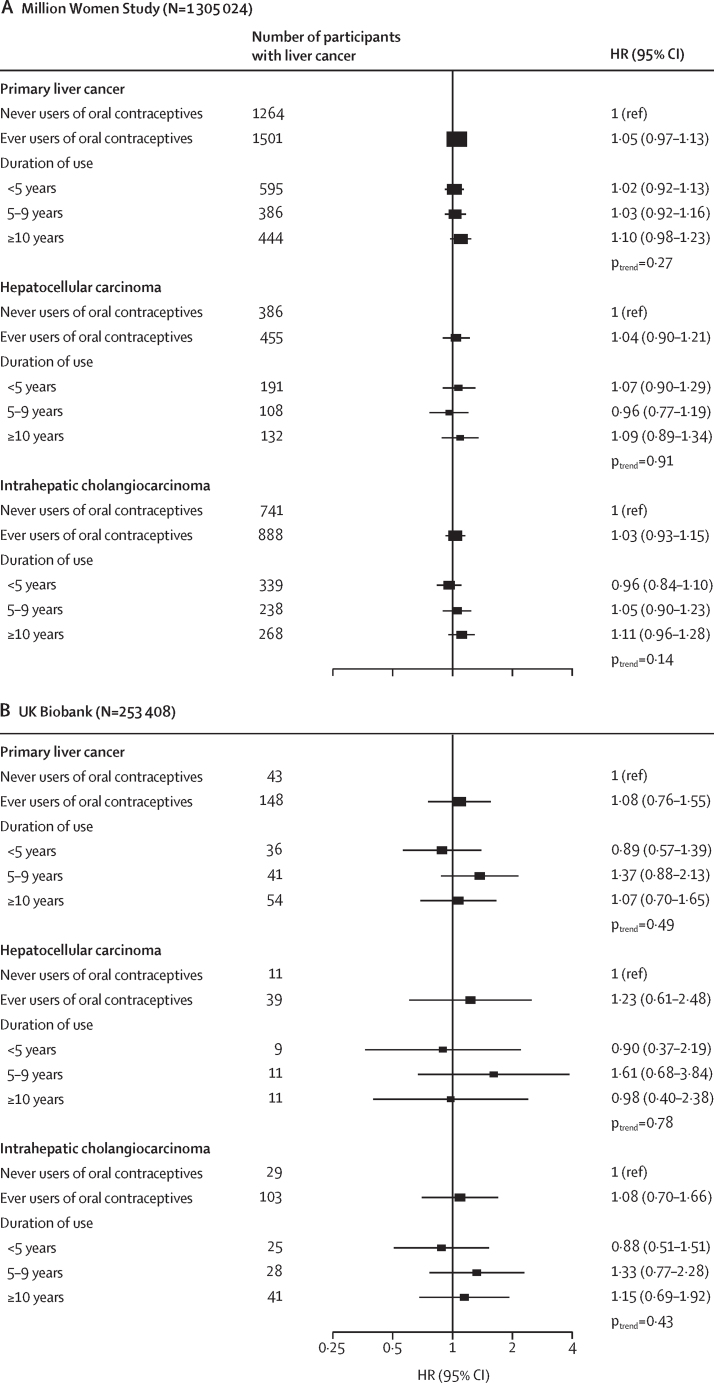
The associations of oral contraceptive use and risk of liver cancer, hepatocellular carcinoma, or intrahepatic cholangiocarcinoma in the Million Women Study (A) and the UK Biobank (B) The Million Women Study analyses were stratified by year of recruitment and year of birth, and adjusted for material deprivation, region of recruitment, BMI, height, physical activity, smoking status, alcohol consumption, education, diabetes status, menopausal status or years since menopause, menopausal hormone therapy use, and history of a hysterectomy. UK Biobank analyses were stratified by age at recruitment, and adjusted for region of recruitment, material deprivation, BMI, height, physical activity, smoking status, alcohol consumption, coffee intake, ethnicity, education, diabetes status, menopausal status and time since menopause, menopausal hormone therapy use, and history of a hysterectomy. Duration of use analyses are included for participants who provided information on duration. p_trends_ were estimated using the median duration for each category, modelling duration as a linear variable, and obtaining the p value from this estimate. Box sizes are proportional to the inverse variance within each category. HR=hazard ratio.

Figure 1 presents the multivariable adjusted associations of oral contraceptive use with primary liver cancer risk; the minimally adjusted estimates are available in the appendix (p 16). Participants who had ever used oral contraceptives did not have a higher risk of liver cancer in comparison with those who had never used oral contraceptives in either the MWS (HR 1·05, 95% CI 0·97–1·13; p=0·27) or the UK Biobank (1·08, 0·76–1·55; p=0·66). Analysis by histological type found no evidence of an association in either MWS (hepatocellular carcinoma HR 1·04, 0·90–1·21; intrahepatic cholangiocarcinoma HR 1·03, 0·93–1·15; p_heterogeneity_=0·92) or in the UK Biobank (hepatocellular carcinoma HR 1·23, 0·61–2·48; intrahepatic cholangiocarcinoma HR 1·08, 0·70–1·66; p_heterogeneity_=0·76). When duration of use was examined, women with the longest duration of use (≥10 years of use) did not have a statistically significant increased risk of liver cancer in either the MWS (HR 1·10, 0·98–1·23; p_trend_=0·27) or the UK Biobank (1·07, 0·70–1·65; p_trend_=0·49; figure 1). For both MWS and UK Biobank, there was no evidence of trend across increasing duration of oral contraceptive use and liver cancer, hepatocellular carcinoma, or intrahepatic cholangiocarcinoma risk (figure 1).

In subgroup analysis looking at differences by age and diabetes status, there was no evidence of heterogeneity in the association of oral contraceptive use with liver cancer risk (appendix p 27). Nor were the findings materially altered after exclusion of the first 4 years of follow-up .

The systematic review database searches identified 7752 records; 3016 duplicate records were removed before screening, and 4406 records were excluded after title and abstract screening. 330 full texts were examined for eligibility, 309 were excluded, and 21 were included in the meta-analysis (appendix p 28). The identified studies were 15 case–control studies^18^, ^19^, ^20^, ^21^, ^22^, ^23^, ^24^, ^25^, ^26^, ^27^, ^28^, ^29^, ^30^, ^31^, ^32^ and six prospective studies^33^, ^34^, ^35^, ^36^, ^37^, ^38^ that assessed the association between oral contraceptive use and liver cancer risk (appendix pp 17–22). The PRISMA flow chart is available in the appendix (p 28). In two instances, we identified articles that included analyses from the same cohort.^39^, ^40^ In our meta-analysis, we used the most up-to-date article^36^ with the exception of when we meta-analysed studies by duration of oral contraceptives use^39^ and when intrahepatic cholangiocarcinoma was the outcome of interest^40^ as the most up-to-date analysis did not include this.

5422 cases of liver cancer were identified across the 23 observational analyses (including the MWS and UK Biobank). The quality assessment scores for case–control and prospective cohort studies are available in the appendix (pp 23–24). Overall, the case–control studies scored lower in quality, with only two case–control studies scoring higher than 6 out of the possible 8 points. All prospective studies except one scored 8 or more out of the possible 9 points on the quality assessment score. In one of the case–control studies,^18^ a crude OR was estimated based on the number of cases and controls in the ever versus never use groups as this was not provided in the text. In one of the prospective studies,^38^ the 95% CIs were estimated based on the unadjusted rate ratio between the number of liver cancer cases and non-cases in the oral contraceptive ever versus never use categories, as the presented 95% CIs were deemed implausible given the observed number of cases. We also presented the premenopausal and postmenopausal estimates separately for this study, as estimates were stratified in this manuscript based on menopausal status at baseline.^38^ In sensitivity analyses we removed this study, and associations remained the same (data not shown).

**Figure 2:**
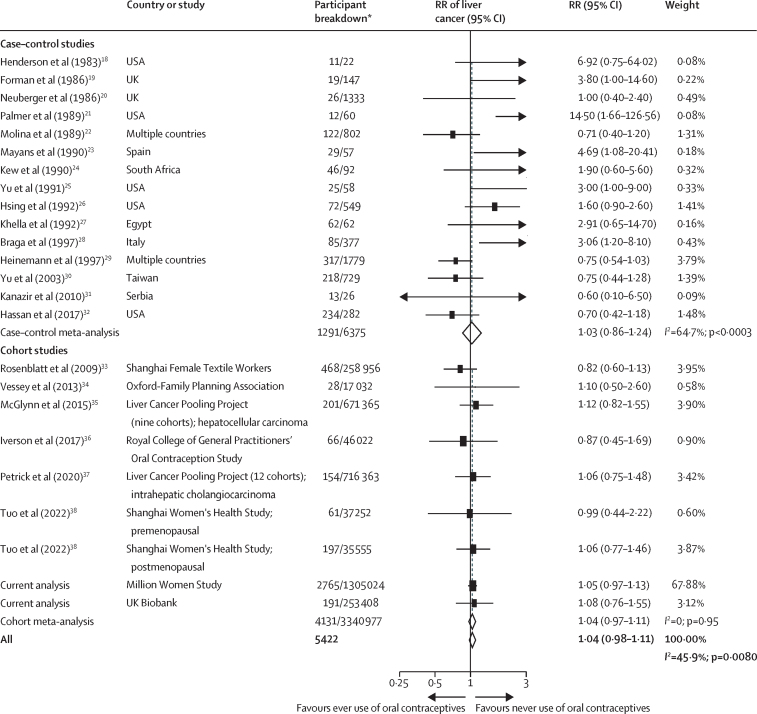
Forest plots for the meta-analysis of observational studies that assessed ever versus never use of oral contraceptives in relation to the risk of liver cancer Box sizes are proportional to the inverse variance within each category. RR=relative risk. *For case–control studies, participant breakdown is liver cancer number of cases/number controls in the study; for cohort studies, participant breakdown is the number of participants who developed liver cancer/total number of participants in the study.

In the meta-analysis comparing the risk of liver cancer in ever use versus never use of oral contraceptives, no association was observed with ever use (RR 1·04, 95% CI 0·98–1·11; figure 2). There was, however, evidence of heterogeneity between studies (*I*^2^=45·9%, p=0·0080), which was largely due to heterogeneity in the estimates obtained from the case–control studies, as there was no evidence of heterogeneity among the prospective cohort study estimates (*I*^2^=0, p=0·95; figure 2). The Egger test showed some suggestion of publication bias (p=0·093), which was attributed to the inclusion of small case–control studies (Egger test for only case–control studies p=0·0008), and funnel plots suggested asymmetry in smaller case–control studies (appendix p 29).

**Figure 3:**
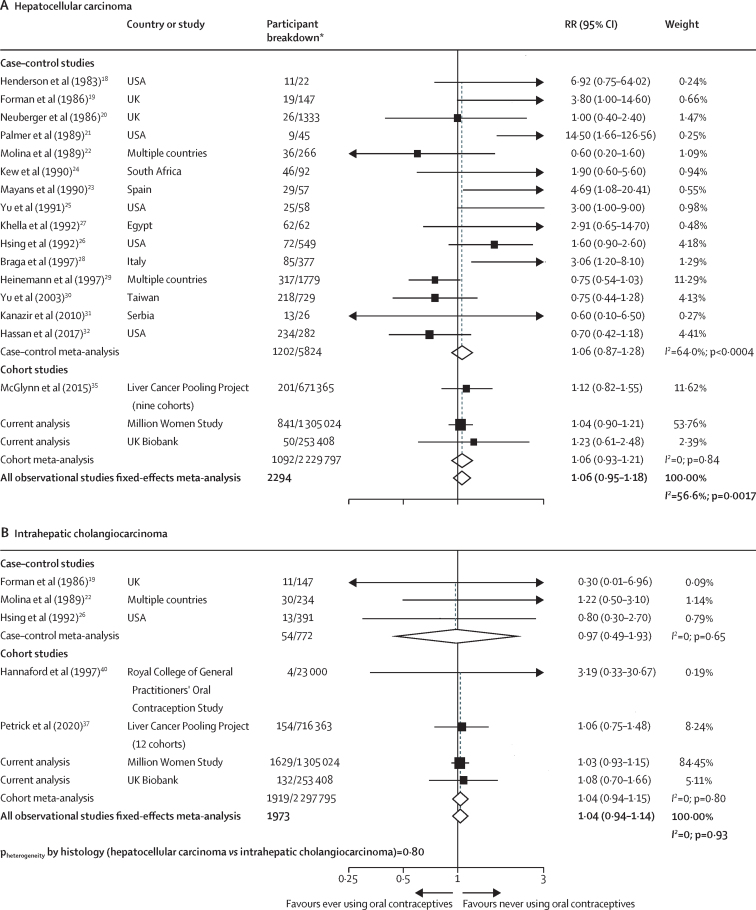
Forest plots for the meta-analysis of all observational studies that assessed ever versus never use of oral contraceptives in relation to risk of hepatocellular carcinoma (A) and intrahepatic cholangiocarcinoma (B) Box sizes are proportional to the inverse variance within each category. RR=relative risk. *For case–control studies, participant breakdown is liver cancer number of cases/number controls in the study; for cohort studies, participant breakdown is the number of liver cancer cases/total number of participants in the study.

When separate meta-analyses were conducted for each of the two main histological subtypes of liver cancer, no associations were observed for ever use versus never use of oral contraceptives. The RR of hepatocellular carcinoma was 1·06 (95% CI 0·95–1·18; *I*^2^=56·6%, p=0·0017); and that of intrahepatic cholangiocarcinoma was 1·04 (0·94–1·14; *I*^2^=0, p=0·93; figure 3). In addition, there was no evidence of variation in the observed associations with ever contraceptive use by histology (p=0·80).

**Figure 4:**
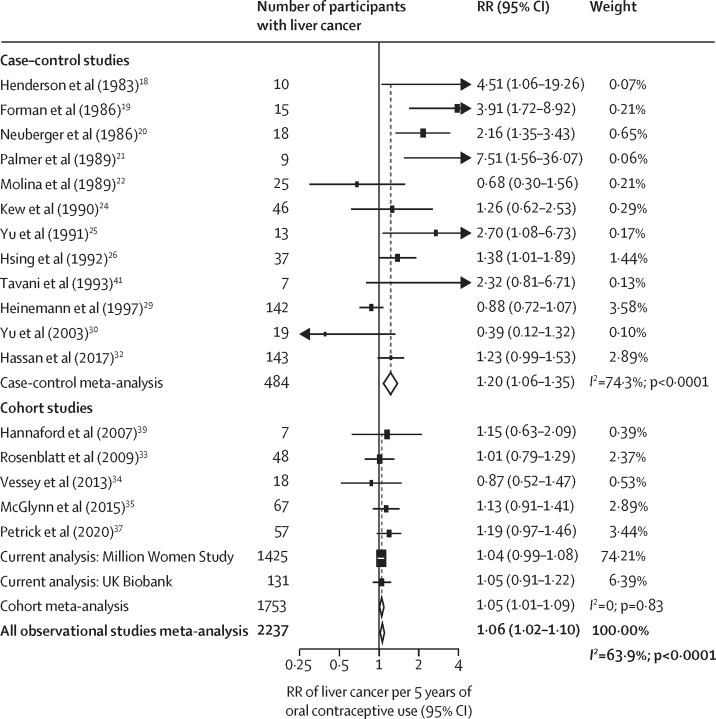
Forest plots for the meta-analysis of all observational studies that assessed duration of use of oral contraceptives (per 5 years of use) in relation to risk of liver cancer Box sizes are proportional to the inverse variance within each category. RR=relative risk.

**Figure 5:**
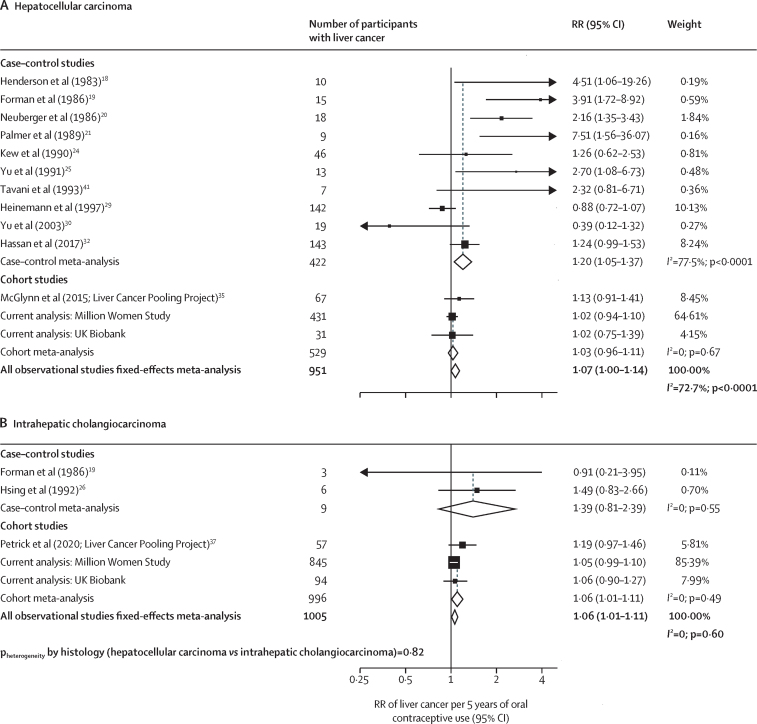
Forest plots for the meta-analysis of all observational studies that assessed duration of use of oral contraceptives (per 5 years of use) in relation to the risk of hepatocellular carcinoma and intrahepatic cholangiocarcinoma Box sizes are proportional to the inverse variance within each category. RR=relative risk.

In meta-analyses that studied duration of oral contraceptive use, there was a slight positive association with total liver cancer risk per 5 years of use (RR 1·06, 95% CI 1·02–1·10; figure 4). However, the RR from the cohort studies (1·05, 1·01–1·09) was lower than for case–control studies (1·20, 1·06–1·35). The Egger test was significant for publication bias (p=0·025) and the funnel plot showed some degree of asymmetry (appendix p 30), which was attributable to the small sized case–control studies included. When associations of duration of use with risk were explored by subtypes of liver cancer, there was no evidence of any difference in this association by subtype (hepatocellular carcinoma RR 1·07, 1·00–1·14; intrahepatic cholangiocarcinoma RR 1·06, 1·01–1·11; p_heterogeneity_=0·82; figure 5). Associations were generally the same when meta-analysing the highest category of duration of use of oral contraceptives with liver cancer risk (appendix p 31) and by liver cancer subtypes (appendix p 32).

## Discussion

In this analysis assessing the association between oral contraceptive use and liver cancer risk, which included over 1·5 million women in the UK, no association between use of oral contraceptives and liver cancer risk was observed in either the MWS or the UK Biobank. When all published observational studies were examined in our meta-analysis, there was no evidence of an association of ever use versus never use of oral contraceptives with total liver cancer risk, or with the risk of either of the two main histological subtypes, hepatocellular carcinoma or intrahepatic cholangiocarcinoma. There was, however, weak evidence of a small increased risk of total liver cancer per 5 years of use of oral contraceptives.

In the most recent IARC monograph on oral contraceptive use, published in 2012, the expert review panel supported the findings of earlier expert review panels^7^, ^9^ that there was evidence that oral contraceptives cause liver cancer.^8^ This position contrasts with the results presented here, which found little to no association of ever use of oral contraceptives with liver cancer risk, although longer duration use (per every 5 years in use) was associated with a slight increase in risk.

The slight increase in risk of liver cancer with increasing duration of use of oral contraceptives was largely driven by the largest cohorts (MWS and UK Biobank; ∼80% of the total weight). However, neither of these studies showed a statistically significant trend with increasing duration of use. Although we carefully adjusted for all measured confounders at recruitment, the possibility of residual confounding or unmeasured confounding variables, particularly the HCV or HBV status of participants, cannot be ruled out. Women might have also developed diabetes over the study period, or they might have underestimated their weight, which are important risk factors for liver cancer.^42^, ^43^ Perfect adjustment for these confounders would possibly have further attenuated the slightly higher association observed for women who used oral contraceptives for a greater duration of time and made this association null. The case–control studies included generally tended to report larger estimates by duration of use; however, these studies are subject to several biases, such as recall bias and control selection bias, which are less prevalent in cohort studies. In the first IARC monograph in 1999, the working group stated that there was sufficient evidence that combined oral contraceptives were associated with hepatocellular carcinoma in the absence of viral exposures. The analyses presented here do not support the hypothesis that an association between oral contraceptives and liver cancer exists in populations in which HBV and HCV are not prevalent. Although oophorectomy has been positively associated with liver cancer risk,^35^ we adjusted for hysterectomy as this procedure is more common, sometimes done with oophorectomy,^44^ and would influence oral contraceptive use and be an important confounder.

In a previous meta-analysis from 2015, which used a random-effects model to combine estimates, a positive association was observed with ever versus never oral contraceptive use in case–control studies, but no association was observed in cohort studies.^45^ In our analyses, we observed no association in all case–control or cohort analyses when examining associations by ever versus never use, probably because we included more studies, had over three-times the number of cases, and used a fixed-effect model, which would have given less weight to findings from small studies as compared with a random-effects model. Heterogeneity was observed for nearly all case–control meta-analyses, probably due to the inconsistent nature of study design, exposure ascertainment, confounder adjustment, and control selection for each individual study. In contrast, in all prospective analyses, there was no evidence of heterogeneity observed between studies across any of the meta-analyses (ie, for all liver cancers, hepatocellular carcinoma, and intrahepatic cholangiocarcinoma).

The formulation of oral contraceptives has changed since they were introduced in 1960^46^ to include lower doses of both oestrogen and progestin. As such, it is possible that earlier formulations of oral contraceptives were associated with increased liver cancer risk. However, when we conducted analyses by age at recruitment (≤55 years and >55 years) to try to approximate women (ie, those aged >55 years) who might have used older formulations of oral contraceptive during the 1960s, there was no evidence of heterogeneity in the results of the MWS or the UK Biobank studies. This sensitivity analysis was a rough estimate to see if there were any differences primarily in the MWS as women would be of reproductive age when the first formulation of oral contraceptive became available. For simplicity and statistical power, women in the UK Biobank were dichotomised the same way, although the cohort was recruited a decade later and therefore these women would have been younger at that time than MWS participants. Similar results showing no association with liver cancer were found in a previous study that separated analyses by oral contraceptive formulations.^29^

Although the overall evidence for an association of oral contraceptives with liver cancer risk, as presented here, was relatively weak, the findings suggest a small association of longer duration use with risk. There are several different mechanisms by which oral contraceptives have been purported to affect risk of liver cancer. Oestrogens have been shown in rat models to induce cholangiocyte proliferation and promote cholangiocarcinogenesis by acting on oestrogen receptor-α and receptor-β.^47^, ^48^, ^49^ In contrast, hepatocytes predominantly express oestrogen receptor-α, and in bile duct ligated rats, overexpression of oestrogen receptor-β in cholangiocytes is observed with a reduction in oestrogen receptor-α in both cholangiocytes and hepatocytes. Epidemiological evidence has also suggested that circulating oestradiol could be positively associated with intrahepatic cholangiocarcinoma in postmenopausal women.^50^ Although hepatocellular carcinomas have also been shown to express oestrogen receptors,^51^ some evidence has suggested that circulating oestrogens could decrease risk of hepatocellular carcinoma in mouse models^52^ or not be associated with hepatocellular carcinoma risk in humans.^50^ Therefore, the pathways by which exogenous oestrogens, progesterone, or both, might influence hepatocarcinogenesis are currently unknown. Even if longer duration of use of oral contraceptives is causally associated with liver cancer risk, based on the RR estimate presented here, and the low incidence of liver cancer observed in women, the absolute risk difference would be very small.

There are numerous strengths to this current analysis. The MWS and UK Biobank are two of the largest existing prospective studies of women. The large sample size, with substantial length of follow-up, is important for attaining sufficient statistical power as liver cancer is uncommon in women (age standardised rate of 6·5 cases per 100 000 women per year).^53^ Moreover, both the MWS and UK Biobank asked detailed information from participants at recruitment about important confounders so that adjustment could be made. These two studies have more than doubled the number of liver cancer cases in comparison with the existing evidence on oral contraceptive use and liver cancer risk, thereby adding substantial power to assess the association. We also used systematic methods to include all existing published observational studies on oral contraceptive use and liver cancer risk to assess the totality of existing evidence.

In addition to the strengths of the study, there were also a few limitations. For both MWS and the UK Biobank, information on HCV or HBV status was not available. However, as the prevalence of infection with HCV or HBV in the UK is relatively low,^54^, ^55^ most participants with liver cancer are unlikely to have been infected with either virus. In the present analysis, as nearly all participants had already undergone menopause, it was not possible to determine whether there was an association between oral contraceptive use and liver cancer in current users or recent past users. However, both hepatocellular carcinoma and intrahepatic cholangiocarcinoma are very uncommon in women of premenopausal age (less than one case per 100 000 women per year).^53^ As most cohorts included in this study were conducted in the UK, the results might not be generalisable to other countries. Although examining liver cancer risk by different formulations of oral contraceptives might have been informative, as has been the case with other cancers, we were unable to do so as the information was not available. However, for around 90% of women in the UK in the late 1980s, combination pills were reported to have been used.^56^ In both the MWS and UK Biobank, oral contraceptive use and duration of use were determined by self-report. As is the case when trying to remember past use of any medication, there might have been some errors in memory, particularly for duration of use. We were also unable to adjust for some potential confounders, such as cirrhosis, as this information was not available at baseline and information on this was only available via hospital admission records, which encompassed very few individuals. Some studies in the meta-analysis included liver and gallbladder cancer as a single combined outcome^34^, ^36^ and did not study subtypes of liver cancer, therefore limiting statistical power for subgroup analysis and potentially introducing noise. However, these studies contributed relatively few participants and any effects should be minimal. Finally, not all studies in the meta-analysis assessed duration of use for oral contraceptives and therefore could not be included in the estimates for that meta-analysis.

In conclusion, there is little evidence to support an association between oral contraceptive use and risk of liver cancer.

### Contributors

### Data sharing

Access to the Million Women Study (MWS) data is through an open access data application. Full details of the MWS data access policy and application process can be viewed online (https://www.ceu.ox.ac.uk/research/the-million-women-study/data-access-and-sharing). UK Biobank is an open access resource. Bona fide researchers can apply to the use the data at https://www.ukbiobank.ac.uk/enable-your-research/register.

## Declaration of interests

We declare no competing interests.
