# Supplementary material for: Oral contraceptive use and risk of liver cancer: a population-based study, systematic review, and meta-analysis
Source: Lancet Oncol. Author manuscript; Available in PMC 2025 Sep 10. (PMC12303860; doi:10.1016/S1470-2045(25)00222-0)
Supplement: Supplementary Material [file EMS207273-supplement-Supplementary_Material.docx]

# Covariate classification

In the Million Women Study, participants with missing information for each covariate were assigned into a missing indicator category. For UK Biobank, we utilised different methods for missing variables due to the smaller number of cases observed in this cohort. If a variable was missing for >5% of the study population, a missing indicator category was utilised. If a continuous variable was missing for <5% of the study population, the median value was used to replace the missing variable. If a categorical variable was missing for <5% of the study population, participants were randomly assigned to categories based on the distribution of the variable in participants that provided information on the variable.

*Region*

In the Million Women Study, participants were grouped into ten broad regions of residence based on the 10 regional cancer registries (9 regions in England – Oxford, East Anglia, South West, Thames, West Midlands, North Yorkshire, Trent, North West (Mersey), North West (Manchester / Lancashire) – and Scotland).

In UK Biobank, participants were grouped into ten broad regions based on the centre at which they were recruited: London (assessment centres: St Bartholomew’s Hospital, Hounslow, Croydon), Wales (assessment centres: Swansea, Wrexham, Cardiff), North-West England (assessment centres: Stockport, Manchester, Liverpool, Bury), North-East England (assessment centres: Newcastle, Middlesbrough), Yorkshire (assessment centres: Leeds, Sheffield), West Midlands (assessment centres: Stoke, Birmingham), East Midlands (assessment centre: Nottingham), South-East England (assessment centres: Oxford, Reading), South-West England (assessment centre: Bristol), Scotland (assessment centres: Glasgow, Edinburgh).

*Body mass index*

Body mass index (BMI) was determined in the Million Women Study from self-reported height and weight at recruitment. From this, BMI was calculated, and women were categorised into the following categories <20, 20–<25, 25–<30, and 30+ kg/m^2^ and an unknown category if information was missing/unknown (~5%). In UK Biobank, participants had their weight and height measured by trained professionals, and from this, were categorised into the following categories: <25, 25.0-29.99, 30.0-34.99, 35-39.99, ≥40 kg/m^2^. Women with missing information (0.51%) were assigned the median value for BMI (26.9 kg/m^2^) and categorized into the respective category.

*Height*

In the Million Women Study, height was self-reported at recruitment and women were categorised into the following categories:<160, 160 to ≤165 and 165+ cm or unknown (1.5% of women). In UK Biobank, height was modelled as a continuous variable per 1 cm increase. Participants with missing information in UK Biobank (0.5%) were assigned the median value 162 cm.

*Physical activity*

In the Million Women Study, women were categorised based on self-reported strenuous activity into the following categories: <once per week, 1-3 times per week and 4+ times per week, with those with missing information assigned to an unknown category (3.6%). In UK Biobank, physical activity was determined from questions on the touchscreen questionnaire which asked about walking, moderate physical activity, and vigorous physical activity. These were used to estimate excess metabolic equivalent (MET)-hours/week of physical activity during work and leisure time. For each of the three activity categories (walking, moderate physical activity and vigorous physical activity), participants were asked how many days in a typical week they did each of the activities for 10 minutes or more. For each category, participants who entered one or more days were then asked how many minutes they spent doing those activities on a typical day. For each activity category, the number of reported days was multiplied by the number of reported minutes on a typical day to generate duration of activity in minutes per week. Activity on a typical day of 1260 min per week (equivalent to an average of 3 hours per day) were truncated at 1260. Total MET values for each category from the International Physical Activity Questionnaire short form were: 3.3 for walking, 4.0 for moderate physical activity and 8.0 for vigorous physical activity. Excess MET values were therefore 2.3 for walking, 3.0 for moderate physical activity and 7.0 for vigorous physical activity. Excess MET-hours per week were calculated by multiplying the excess MET value for each activity by the duration of activity in hours per week. Participants with missing information (5.4%) were categorized into a ‘missing’ category.

*Smoking status*

In the Million Women Study, smoking status was categorised as never, former, current <15 cigarettes a day and current 15+ cigarettes a day based on responses from the recruitment questionnaire. If smoking status could not be determined, participants were categorised into a missing category (5.7% of participants). In UK Biobank, smoking status was categorised as never, former, or current smoker and was determined from questions from the recruitment questionnaire. Participants were asked “Do you smoke tobacco now?” and “in the past, how often have you smoked tobacco?” to determine their smoking status. Participants with missing information on smoking status (0.6%) were randomly assigned into one of the three categories.

*Alcohol consumption*

In the Million Women Study, alcohol intake was determined based on the number of drinks women had per week. From this, women were categorised as non-drinkers (< 1per week), 1-2.5 drinks per week, 3-6.5 drinks per week, 7-14.5 drinks per week, and 15+ drinks per week or unknown/missing (0.7% of participants). In UK Biobank, women were asked on the baseline questionnaire at recruitment how often they drank alcohol with the possible responses being: “daily or almost daily”, “three or four times a week”, “once or twice a week”, “one to three times a month”, “special occasions only”, “never”, or “prefer not to answer”. Participants were then asked about their weekly or monthly intake of pints of beer, glasses of red wine, glasses of white wine/champagne, glasses of fortified wine, measures of spirits/liqueurs and glasses of other alcohol. A pint of beer was assumed to contain 20 grams of alcohol, and all other drinks contained 10 grams of alcohol. We then summed their total weekly or monthly consumption of alcohol accordingly. If the participant reported “do not know” or “prefer not to answer” to one of these questions on weekly or monthly consumption, they were coded as missing, except for “other alcohol”, in which case we assigned them 0 grams from other alcohol. We used participants reported weekly consumption of alcohol. If this was unknown, due to the participant reporting they only drank alcohol one to three times months or on special occasions, we used monthly consumption, if available. To get an estimated daily total, we divided weekly consumption by 7 (or monthly consumption by 30.4375). Alcohol consumption was categorised as <1 g/day, 1-9.99 g/day, 10-19.99 g/day, and ≥20 g/day, never or unknown. For participants who had unknown grams/day of alcohol but who reported consuming alcohol intake on “special occasions”, we assigned them to the category of “<1 g/day”. A total of 1.61% of participants had missing information and we replaced their missing value with mean intake (18.1 g/day) and categorized these participants accordingly.

*Deprivation Index*

In both the Million Women Study and UK Biobank, the Townsend deprivation index was based on the preceding national census output areas. Each participant was assigned a score in correspondence to the output area in which their postcode was located. From this, participants were categorised into quintiles from most deprived to least deprived. Participants with missing information in UK Biobank (0.1%) were randomly assigned into one of the 5 quintiles whereas in the Million Women Study participants were categorised into a missing category (0.7% of participants).

*Education*

In the Million Women Study, participants were categorized into the following categories: no educational qualifications, technical educational qualifications, secondary educational qualifications and tertiary educational qualifications based on answers to questions on the recruitment questionnaire. In UK Biobank, participants were asked ‘Which of the following qualification do you have?’ being able to select more than one. Possible answers were: College or University degree; A levels/AS levels or equivalent; O levels/GCSEs or equivalent; CSEs or equivalent; NVQ or HND or HNC or equivalent; Other professional qualifications example: nursing, teaching; None of the above; Prefer not to answer. We grouped participants into the following categories, based on their highest reported level of education: College or University degree, vocational qualifications (other professional qualifications/NVQ or HND or HNC), optional national exams at ages 17 to 18 years (A levels/AS levels), national exams at age 16 years (O levels/GCSEs/CSEs), none of the above, prefer not to answer, and missing (unknown/missing)).

*Coffee intake*

In the Million Women Study, coffee intake was not asked on the baseline questionnaire and therefore was not adjusted for. In UK Biobank, women were asked at recruitment how many cups of regular coffee they drink each day. From their responses we categorised participants as none, 1 cup/day, 2 cups/day, 3-4 cups/day, and 5+ cups a day. Around 7.56% of participants did not answer this question so they were assigned into a missing category variable.

*Ethnicity*

In the Million Women Study, women were not asked about their ethnicity on the baseline questionnaire and therefore this was not adjusted for. In follow-up questionnaires, ethnicity was asked at followed up and the large majority of women reported that they were White (96%), therefore adjustment for ethnicity is unlikely to influence these results. In UK Biobank, ethnicity of participants was determined from questions in the touchscreen questionnaire “What is your ethnic group?”. Options included: White, Mixed race or other, Asian or Asian British, Black or Black British, Chinese, and other ethnic group. From this, women were categorised into two groups, white or non-white participants. Participants with missing information (0.5%) were randomly assigned based on distribution of participants to categories.

*Diabetes status*

In the Million Women Study, living with diabetes was established based on participants’ self-reported diagnosis of, or treatment for, diabetes at recruitment (in response to the questions: “Have you ever been diagnosed with diabetes” or “Are you now being treated for diabetes”). From this, participants were categorised into yes, no, or unknown (0.09%). In UK Biobank, participants’ diabetes status was determined using multiple questions from recruitment. First, from the question “Has a doctor ever told you that you have diabetes?” participants were classified as “yes”, “no” or “unknown” based on their response. As well, participants who reported using metformin or insulin at recruitment were considered to have diabetes and included in the “yes” category. Finally, if a participant had a measured glycated hemoglobin (HbA1c) of ≥ 48 mmol/mol at recruitment, they were defined as having diabetes and included in the “yes” category. Participants with missing or unknown information on diabetes status (<0.5%) were randomly assigned to categories.

*Menopausal status*

We determined menopausal status in the Million Women Study based on questions asked on the baseline questionnaire and from this, women were categorised as pre/perimenopausal, postmenopausal with time since menopause at recruitment specified as <5 years, 5-9 years, or 10+ years at study entry, postmenopausal but time since unknown, or as having unknown menopausal status (15.6% of participants). If women were deemed pre- or peri-menopausal at the time of recruitment, we entered them into the risk set when they turned 55 years of age as 99% of women will have undergone menopause by this time.

In the UK Biobank, women were categorised into premenopausal, postmenopausal: based on time since menopause <5 years, 5-9 years, or 10+ years at study entry, or unknown. Menopausal status was first determined by multiple questions asked on the baseline questionnaire and females were defined as being postmenopausal if they:

- Answered “yes” to having gone through menopause
- Answered “not sure” or did not answer if they had gone through menopause and:
  - were ≥55 years of age, or
  - had a bilateral oophorectomy

Women were defined as being pre-menopausal if they:

- Answered ‘no’ to the question regarding having gone through menopause, or
- Reported they were ‘not sure’ or did not respond to if they had gone through menopause and:
  - Were <50 years of age, did not have a bilateral oophorectomy/hysterectomy, and reported they were not using menopausal hormone therapy.
  - Were <50 years of age, reported they were menstruating today, and did not have a bilateral oophorectomy/hysterectomy.

All other women that did not fit into these two categories were categorised as unknown.

In UK Biobank, women who were not postmenopausal at recruitment were excluded from the analysis.

*Oral contraceptive use*

In both the MWS and UK Biobank, women were asked if they had ever taken oral contraceptives before. In UK Biobank, some participants reported that they were currently taking oral contraceptives. Based on responses to this question women were categorised into “ever” taken oral contraceptives and “never” with an unknown category if this information was not provided.

*Hysterectomy*

In the Million Women Study, having a hysterectomy was determined from self-report and from hospital admission records (with OPCS4 codes Q07 and Q08) before study recruitment (for the small proportion of women with hospital records before recruitment). In UK Biobank, having a hysterectomy was only determined from self-report. From this, women were categorised as “no”, “yes”, or” unknown”. In total in UK Biobank, 11.5% of participants were listed as unknown/missing and a missing category was utilised, whereas 0.6% of participants in the Million Women Study had unknown hysterectomy status.

# Systematic Review and Meta-analysis

The systematic review and meta-analysis was registered with PROSPERO (PROSPERO Number: CRD42024552518).

*Literature search*

A research librarian at the National Cancer Institute carried out the search of the literature for published articles that assessed the association of exogenous hormone use and liver cancer risk from all observational studies. Searching was conducted from database inception to June 28^th^, 2024. The following databases were searched: Embase, PubMed/MEDLINE, CINAHL, Scopus, and Web of Science for observational studies examining the exogenous hormone use with liver cancer risk. Supplementary Table 1 presents the search strategies employed across databases. Once titles and abstracts were obtained, two independent researchers (CZW, AW), using Covidence web-based systematic review screening software (https://app.covidence.org/), independently screened records for eligibility and selected abstracts to be fully reviewed. Once abstracts were obtained, full texts of articles were searched, and reviewers made decisions whether to include or exclude. Any discrepancies were discussed between the reviewers until a consensus was reached. If no consensus was reached, a third reviewer (KAM) made the final decision.

*Study selection*

The inclusion criteria for the studies were: 1) observational studies, 2) presented in English, 3) studies that assessed the association between menopausal hormone therapy with liver cancer (all or any subtype of liver cancer), and 4) studies that reported hazard ratios, odds ratios, or relative risks (RR) with 95% confidence intervals or presented the cases/controls within each category in which we could estimate odds ratios and 95% confidence intervals. We excluded any studies that were cross-sectional, did not assess liver cancer risk, did not look at menopausal hormone therapy use (e.g., intrauterine contraceptives), or the main text was not available in English.

*Data extraction*

From the identified studies that met the eligibility criteria, one author extracted the study information including information on RR and 95% CI within each category of menopausal hormone therapy (ever vs. never) as well as for categories of duration of menopausal hormone therapy use with risk of liver cancer. We also extracted the last name of author, publication year, country, cohort name (if applicable), sample size, mean age at entry, mean/median follow-up time (for cohort studies), total number of cases, number of cases and participants within each category, outcome ascertainment, and covariates adjusted for.

*Risk of bias*

The Newcastle-Ottawa Quality Assessment Scale for case-control studies and prospective studies was used to assess quality of studies. Specifically, two authors (CZW, AW) assessed the risk of bias using a slightly adapted Newcastle-Ottawa Quality Assessment Scale separately for the case-control studies and prospective studies. Case-control studies that scored ≥6 out of 8 were considered of high quality, whereas prospective studies that scored at least 8 out of 9 were considered high quality.^2^

*Menopausal hormone therapy* *duration of use meta-analysis*

For articles that assessed associations between menopausal hormone therapy duration and liver cancer risk, we obtained the duration of use categories used, number of cases/controls/participants in each category, and relative risks, to determine associations per 5 years of use in a dose-response meta-analysis. If the number of controls was not presented, we replaced this with the same number of cases. If the number of participants from cohort studies was not presented, we divided the number of people in the cohort by the number of categories. For each of the categories presented with a range, we replaced this with the middle value. For example, if a study presented categories of menopausal hormone therapy use by never, <1 years, 1 to <3 years, 3 to <6 years, 6 to <8 years, and 8+ years, we replaced these categories to 0 years, 0.5 years, 2 years, 4.5 years, 7 years, and 11 years, respectively. For the highest category, duration of use was approximated by adding 3 years to the cut off of the category in order to approximate the median duration. This was based on the duration of use in Million Women Study observed among the highest category (13 years for women in the 10+ years of use category). Using generalized least squares, we estimated associations per 5 years of menopausal hormone therapy use in each of these studies. A fixed effects meta-analysis was used to combine these estimates to determine associations per 5 years of menopausal hormone therapy use.

# Supplementary Tables

**Supplementary Table 1.** Search strategy for systematic literature review.

| Database: PubMed/MEDLINE  Platform: National Library of Medicine | | |
| --- | --- | --- |
|  | Concept | Search Strategy |
| #1 | Exogenous Hormone | "[Hormone Replacement Therapy"[Mesh](https://www.ncbi.nlm.nih.gov/mesh/68020249)] OR "hormon* replacement therap*"[Title/Abstract] OR "menopausal hormone therapy"[Title/Abstract:~4] OR "menopausal hormone therapies"[Title/Abstract:~4] OR "menopausal hormonal treatment"[Title/Abstract:~4] OR "menopausal hormonal treatments"[Title/Abstract:~4] OR "hormone substitution"[Title/Abstract] OR (("[Postmenopause"[Mesh]](https://www.ncbi.nlm.nih.gov/mesh/?term=postmenopause) OR "postmenopaus*"[Title/Abstract] OR "post-menopaus*"[Title/Abstract]) AND ("hormon* treatment*"[Title/Abstract] OR "hormon* therap*"[Title/Abstract])) OR "estrogen replacement"[Title/Abstract:~6] OR "estrogen therapy"[Title/Abstract:~6] OR "estrogen therapies"[Title/Abstract:~6] OR "oestrogen replacement"[Title/Abstract:~6] OR "oestrogen therapy"[Title/Abstract:~6] OR "oestrogen therapies"[Title/Abstract:~6] OR "oestrogen treatment"[Title/Abstract:~6] OR "estrogen treatment"[Title/Abstract:~6] OR "estrogen treatments"[Title/Abstract:~6] OR "exogenous hormon*"[Title/Abstract] OR ["Contraceptives, Oral"[Mesh]](https://www.ncbi.nlm.nih.gov/mesh/68003276) OR "oral contracept*"[Title/Abstract] OR ["Estradiol"[Mesh](https://www.ncbi.nlm.nih.gov/mesh/68004958)] OR "estradiol*"[Title/Abstract] OR "[Spironolactone"[Mesh]](https://www.ncbi.nlm.nih.gov/mesh/68013148) OR "spironolactone*"[Title/Abstract] OR "[Cyproterone Acetate"[Mesh]](https://www.ncbi.nlm.nih.gov/mesh/68017373) OR "cyproterone acetate*"[Title/Abstract] OR ["Azasteroids"[Mesh](https://www.ncbi.nlm.nih.gov/mesh/68001378)] OR "azasteroid*"[Title/Abstract] OR "finasteride*"[Title/Abstract] OR "dutasteride*"[Title/Abstract] OR ["Leuprolide"[Mesh]](https://www.ncbi.nlm.nih.gov/mesh/?term=leuprolide) OR "leuprolide*"[Title/Abstract] OR "[Goserelin"[Mesh]](https://www.ncbi.nlm.nih.gov/mesh/?term=goserelin) OR "goserelin*"[Title/Abstract] OR ["histrelin" [Supplementary Concept]](https://www.ncbi.nlm.nih.gov/mesh/?term=histrelin) OR "histrelin*"[Title/Abstract] OR ["Progesterone"[Mesh]](https://www.ncbi.nlm.nih.gov/mesh/68011374) OR "progesterone*"[Title/Abstract] OR ["Progestins"[Mesh]](https://www.ncbi.nlm.nih.gov/mesh/68011372) OR "progestin*"[Title/Abstract] |
| #2 | Liver Cancer | ["Liver Neoplasms"[Mesh](https://www.ncbi.nlm.nih.gov/mesh/68008113)] OR "liver cancer"[Title/Abstract:~4] OR "liver cancers"[Title/Abstract:~4] OR "liver tumo*"[Title/Abstract] OR "hepat* cancer*"[Title/Abstract] OR "hepat* neoplasm*"[Title/Abstract] OR "hepat* carcinoma*"[Title/Abstract] OR "hepat* malignan*"[Title/Abstract] OR "hepat* tumor*"[Title/Abstract] OR "hepat* tumour*"[Title/Abstract] OR "hepat* adenoma*"[Title/Abstract] OR "intrahepatic cholangiocarcinoma*"[Title/Abstract] OR "intra-hepatic cholangiocarcinoma*"[Title/Abstract] OR ((["Neoplasms"[Mesh]](https://www.ncbi.nlm.nih.gov/mesh/68009369) OR "neoplasm*"[Title/Abstract] OR "[Carcinoma"[Mesh]](https://www.ncbi.nlm.nih.gov/mesh/68002277) OR "carcinoma*"[Title/Abstract] OR "malignan*"[Title/Abstract] OR "adenocarcinoma*"[Title/Abstract] OR "adenoma*"[Title/Abstract] OR "sarcoma*"[Title/Abstract] OR "metastasis"[Title/Abstract] OR "metastases"[Title/Abstract] OR "tumor"[Title/Abstract] OR "tumors"[Title/Abstract] OR "tumour*"[Title/Abstract]) AND ("[Liver"[Mesh]](https://www.ncbi.nlm.nih.gov/mesh/68008099) OR ["Liver Diseases"[Mesh Terms:noexp](https://www.ncbi.nlm.nih.gov/mesh/68008107)] OR "liver"[Title/Abstract])) |
| #3 | Limits: Human & publication type | ((#1 AND #2) NOT ("Animals"[Mesh] NOT ("Animals"[Mesh] AND "Humans"[Mesh]))) NOT ("editorial"[Publication Type] OR "comment"[Publication Type] OR "news"[Publication Type] OR "letter"[Publication Type] OR "Case Reports as Topic"[Mesh] OR "Case Reports"[Publication Type] OR "retracted publication"[Publication Type] OR "retraction of publication"[Publication Type] OR "retraction of publication"[Title/Abstract] OR "retraction notice"[Title] OR "retracted publication"[Title] OR "Congress"[Publication Type] OR "Consensus Development Conference"[Publication Type] OR "conference abstract*"[Title/Abstract] OR "conference proceeding*"[Title/Abstract] OR "conference paper*"[Title/Abstract] OR "proceeding*"[Title]) Filters: English |

| Database: Embase  Platform: Elsevier | | |
| --- | --- | --- |
|  | Concept | Search Strategy |
| #1 | Exogenous Hormone | 'hormone substitution'/exp OR 'hormon* replacement therap*':ti,ab,kw OR (('menopausal' NEAR/4 'hormone therap*'):ti,ab,kw) OR (('menopausal' NEAR/4 'hormonal treatment*'):ti,ab,kw) OR 'hormone substitution':ti,ab,kw OR (('postmenopause'/exp OR 'postmenopaus*':ti,ab,kw OR 'post-menopaus*':ti,ab,kw) AND ('hormon* treatment*':ti,ab,kw OR 'hormon* therap*':ti,ab,kw)) OR ((estrogen NEAR/6 replacement):ti,ab,kw) OR ((estrogen NEAR/6 therap*):ti,ab,kw) OR ((oestrogen NEAR/6 replacement*):ti,ab,kw) OR ((oestrogen NEAR/6 therap*):ti,ab,kw) OR ((oestrogen NEAR/6 treatment*):ti,ab,kw) OR ((estrogen NEAR/6 treatment*):ti,ab,kw) OR 'exogenous hormon*':ti,ab,kw OR 'oral contraceptive agent'/exp OR 'oral contracept*':ti,ab,kw OR 'estradiol'/exp OR 'estradiol*':ti,ab,kw OR 'spironolactone'/exp OR 'spironolactone*':ti,ab,kw OR 'cyproterone acetate'/exp OR 'cyproterone acetate*':ti,ab,kw OR 'azasteroid'/exp OR 'azasteroid*':ti,ab,kw OR 'finasteride*':ti,ab,kw OR 'dutasteride*':ti,ab,kw OR 'leuprorelin'/exp OR 'leuprolide*':ti,ab,kw OR 'goserelin'/exp OR 'goserelin*':ti,ab,kw OR 'histrelin*':ti,ab,kw OR 'progesterone'/exp OR 'progesterone*':ti,ab,kw OR 'progestin*':ti,ab,kw |
| #2 | Liver Cancer | 'liver tumor'/exp OR 'liver tumo*':ti,ab,kw OR ((liver NEAR/4 cancer*):ti,ab,kw) OR 'hepat* cancer*':ti,ab,kw OR 'hepat* neoplasm*':ti,ab,kw OR 'hepat* carcinoma*':ti,ab,kw OR 'hepat* malignan*':ti,ab,kw OR 'hepat* tumor*':ti,ab,kw OR 'hepat* tumour*':ti,ab,kw OR 'hepat* adenoma*':ti,ab,kw OR 'intrahepatic cholangiocarcinoma*':ti,ab,kw OR 'intra-hepatic cholangiocarcinoma*':ti,ab,kw OR (('neoplasm'/exp OR 'neoplasm*':ti,ab,kw OR 'carcinoma'/exp OR 'carcinoma*':ti,ab,kw OR 'malignan*':ti,ab,kw OR 'adenocarcinoma*':ti,ab,kw OR 'adenoma*':ti,ab,kw OR 'sarcoma*':ti,ab,kw OR 'metastasis':ti,ab,kw OR 'metastases':ti,ab,kw OR 'tumor':ti,ab,kw OR 'tumors':ti,ab,kw OR 'tumour*':ti,ab,kw) AND ('liver'/exp/mj OR 'liver disease'/exp/mj OR 'liver':ti,ab,kw)) |
| #3 | Limits: Human & publication type | #1 AND #2 AND ([article]/lim OR [article in press]/lim) AND [english]/lim NOT ([animals]/lim NOT ([animals]/lim AND [humans]/lim)) NOT ([conference abstract]/lim OR [conference paper]/lim OR [conference review]/lim OR 'conference paper'/exp OR 'conference abstract*':ab,ti OR 'conference proceeding*':ab,ti OR 'proceeding*':ti OR [editorial]/lim OR 'editorial'/exp OR 'retraction notice'/exp OR 'retraction'/exp OR 'retraction of publication':ab,ti OR 'retraction notice':ti OR 'retracted publication':ab,ti OR [letter]/lim OR [note]/lim OR 'case report'/exp OR 'case report':ti) |

###

| Database: CINAHL Plus  Platform: EBSCOhost | | |
| --- | --- | --- |
|  | Concept | Search Strategy |
| #1 | Exogenous Hormone | ( (MH "Hormone Replacement Therapy") OR (MH "Contraceptives, Oral") OR (MH "Estradiol") OR (MH "Spironolactone") OR (MH "Leuprolide") OR (MH "Goserelin") OR (MH "Progesterone") ) OR TI ( "hormon* replacement therap*" OR (menopausal N4 "hormone therap*") OR (menopausal N4 "hormonal treatment*") OR "hormone substitution" OR (estrogen N6 replacement*) OR (estrogen N6 therap*) OR (oestrogen N6 replacement*) OR (oestrogen therap*) OR (oestrogen N6 treatment*") OR (estrogen N6 treatment*) OR "exogenous hormon*" OR "oral contracept*" OR "estradiol*" OR "spironolactone*" OR "cyproterone acetate*" OR "azasteroid*" OR "finasteride*" OR "dutasteride*" OR "leuprolide*" OR "goserelin*" OR "histrelin*" OR "progesterone*" OR "progestin*" OR (((MH "Postmenopause") OR "postmenopaus*" OR "post-menopaus*") AND ("hormon* treatment*" OR "hormon* therap*")) ) OR AB ( "hormon* replacement therap*" OR (menopausal N4 "hormone therap*") OR (menopausal N4 "hormonal treatment*") OR "hormone substitution" OR (estrogen N6 replacement*) OR (estrogen N6 therap*) OR (oestrogen N6 replacement*) OR (oestrogen therap*) OR (oestrogen N6 treatment*") OR (estrogen N6 treatment*) OR "exogenous hormon*" OR "oral contracept*" OR "estradiol*" OR "spironolactone*" OR "cyproterone acetate*" OR "azasteroid*" OR "finasteride*" OR "dutasteride*" OR "leuprolide*" OR "goserelin*" OR "histrelin*" OR "progesterone*" OR "progestin*" OR (((MH "Postmenopause") OR "postmenopaus*" OR "post-menopaus*") AND ("hormon* treatment*" OR "hormon* therap*")) ) |
| #2 | Liver Cancer | (MH "Liver Neoplasms+") OR TI ( (liver N4 cancer*) OR "hepat* cancer*" OR "hepat* neoplasm*" OR "hepat* carcinoma*" OR "hepat* malignan*" OR "hepat* tumor*" OR "hepat* tumour*" OR "hepat* adenoma*" OR "intrahepatic cholangiocarcinoma*" OR "intra-hepatic cholangiocarcinoma*" OR (((MH "Neoplasms") OR (MH "Carcinoma") OR "neoplasm*" OR "carcinoma*" OR "malignan*" OR "adenocarcinoma*" OR "adenoma*" OR "sarcoma*" OR "metastasis" OR "metastases" OR "tumor" OR "tumors" OR "tumour*") AND ((MH "Liver") OR (MH "Liver Diseases")OR "liver")) ) OR AB ( (liver N4 cancer*) OR "hepat* cancer*" OR "hepat* neoplasm*" OR "hepat* carcinoma*" OR "hepat* malignan*" OR "hepat* tumor*" OR "hepat* tumour*" OR "hepat* adenoma*" OR "intrahepatic cholangiocarcinoma*" OR "intra-hepatic cholangiocarcinoma*" OR (((MH "Neoplasms") OR (MH "Carcinoma") OR "neoplasm*" OR "carcinoma*" OR "malignan*" OR "adenocarcinoma*" OR "adenoma*" OR "sarcoma*" OR "metastasis" OR "metastases" OR "tumor" OR "tumors" OR "tumour*") AND ((MH "Liver") OR (MH "Liver Diseases")OR "liver")) ) |
| #3 | Limits: Human & publication type | S1 AND S2 NOT ((MH "Animals+") OR (MH "Animal Studies")) NOT ((MH "Retracted Publication") OR (MH "Retraction of Publication) OR (MH "Congresses and Conferences")OR (MH "Case Studies")) Limiters - Peer Reviewed; English Language; Expanders - Apply equivalent subjects; Search modes - Proximity |

| Database: Web of Science (Core Collection)  Platform: Clarivate Analytics | | |
| --- | --- | --- |
|  | Concept | Search Strategy |
| #1 | Exogenous Hormone | TS=("hormon* replacement therap*" OR (menopausal NEAR/4 "hormone therap*") OR (menopausal NEAR/4 "hormonal treatment*") OR "hormone substitution*" OR (( "postmenopaus*" OR "post-menopaus*") AND ("hormon* treatment*" OR "hormon* therap*")) OR (estrogen NEAR/6 replacement*) OR (estrogen NEAR/6 therap*) OR (oestrogen NEAR/6 replacement*) OR (oestrogen NEAR/6 therap*) OR (oestrogen NEAR/6 treatment*) OR (estrogen NEAR/6 treatment*) OR "exogenous hormon*" OR "oral contracept*" OR "estradiol*" OR "spironolactone*" OR "cyproterone acetate*" OR "azasteroid*" OR "finasteride*" OR "dutasteride*" OR "leuprolide*" OR "goserelin*" OR "histrelin*" OR "progesterone*" OR "progestin*" ) |
| #2 | Liver Cancer | TS=((liver NEAR/4 cancer*) OR "hepat* cancer*" OR "hepat* neoplasm*" OR "hepat* carcinoma*" OR "hepat* malignan*" OR "hepat* tumor*" OR "hepat* tumour*" OR "hepat* adenoma*" OR "intrahepatic cholangiocarcinoma*" OR "intra-hepatic cholangiocarcinoma*" OR ("liver" AND ("neoplasm*" OR "carcinoma*" OR "malignan*" OR "adenocarcinoma*" OR "adenoma*" OR "sarcoma*" OR "metastasis" OR "metastases" OR "tumor" OR "tumors" OR "tumour*")) ) |
| #3 | Limits: Human & publication type | #2 AND #1 and Preprint Citation Index (Exclude – Database) and Animals  (Exclude – MeSH Headings) and Humans (MeSH Headings) and Case Report or Abstract or Meeting or Letter or Editorial Material or Book or Reference Material or News (Exclude – Document Types) and English (Languages) and Web of Science Core Collection (Database) |

| **Scopus** | | |
| --- | --- | --- |
|  | Concept | Search Strategy |
| #1 | Exogenous Hormone | (TITLE-ABS-KEY ("hormon* replacement therap*" OR (menopausal W/4 "hormone therap*") OR (menopausal W/4 ‘hormonal AND treatment*") OR " hormone AND substitution* " OR (( " postmenopaus* " OR " post-menopaus* ") AND (" hormon* AND treatment* " OR " hormon* AND therap* ")) OR (estrogen W/6 replacement*) OR (estrogen W/6 therap*" ) OR ( oestrogen W/6 replacement* ) OR ( oestrogen W/6 therap* ) OR ( oestrogen W/6 treatment* ) OR ( estrogen W/6 treatment* ) OR "exogenous hormon*" OR "oral contracept*" OR "estradiol*" OR "spironolactone*" OR "cyproterone acetate*" OR "azasteroid*" OR "finasteride*" OR "dutasteride*" OR "leuprolide*" OR "goserelin*" OR "histrelin*" OR "progesterone*" OR "progestin*" ) |
| #2 | Liver Cancer | TITLE-ABS-KEY ( ( liver W/4 cancer* ) OR "hepat* cancer*" OR "hepat* neoplasm*" OR "hepat* carcinoma*" OR "hepat* malignan*" OR "hepat* tumor*" OR "hepat* tumour*" OR "hepat* adenoma*" OR "intrahepatic cholangiocarcinoma*" OR "intra-hepatic cholangiocarcinoma*" OR ( "liver" AND ( "neoplasm*" OR "carcinoma*" OR "malignan*" OR "adenocarcinoma*" OR "adenoma*" OR "sarcoma*" OR "metastasis" OR "metastases" OR "tumor" OR "tumors" OR "tumour*" ) ) ) AND NOT TITLE-ABS-KEY ( "editorial" OR "letter" OR "case report*" OR "retracted publication" OR "conference abstract*" OR "conference proceeding*" OR "conference paper*" ) |
| #3 | Limits: Human & publication type | NOT TITLE-ABS-KEY ( "editorial" OR "letter" OR "case report*" OR "retracted publication" OR "conference abstract*" OR "conference proceeding*" OR "conference paper*" ) AND NOT TITLE ( "retracted publication" OR "retraction notice" OR proceeding* ) ) |
| #4 |  | ( TITLE-ABS-KEY ( "hormon* replacement therap*" OR ( menopausal W/4 "hormone therap*" ) OR ( menopausal W/4 ‘hormonal AND treatment* ") OR " hormone AND substitution* " OR (( " postmenopaus* " OR " post-menopaus* ") AND (" hormon* AND treatment* " OR " hormon* AND therap* ")) OR (estrogen W/6 replacement*) OR (estrogen W/6 therap*" ) OR ( oestrogen W/6 replacement* ) OR ( oestrogen W/6 therap* ) OR ( oestrogen W/6 treatment* ) OR ( estrogen W/6 treatment* ) OR "exogenous hormon*" OR "oral contracept*" OR "estradiol*" OR "spironolactone*" OR "cyproterone acetate*" OR "azasteroid*" OR "finasteride*" OR "dutasteride*" OR "leuprolide*" OR "goserelin*" OR "histrelin*" OR "progesterone*" OR "progestin*" ) AND TITLE-ABS-KEY ( ( liver W/4 cancer* ) OR "hepat* cancer*" OR "hepat* neoplasm*" OR "hepat* carcinoma*" OR "hepat* malignan*" OR "hepat* tumor*" OR "hepat* tumour*" OR "hepat* adenoma*" OR "intrahepatic cholangiocarcinoma*" OR "intra-hepatic cholangiocarcinoma*" OR ( "liver" AND ( "neoplasm*" OR "carcinoma*" OR "malignan*" OR "adenocarcinoma*" OR "adenoma*" OR "sarcoma*" OR "metastasis" OR "metastases" OR "tumor" OR "tumors" OR "tumour*" ) ) ) AND NOT TITLE-ABS-KEY ( "editorial" OR "letter" OR "case report*" OR "retracted publication" OR "conference abstract*" OR "conference proceeding*" OR "conference paper*" ) AND NOT TITLE ( "retracted publication" OR "retraction notice" OR proceeding* ) ) AND ( LIMIT-TO ( EXACTKEYWORD , "Human" ) OR LIMIT-TO ( EXACTKEYWORD , "Humans" ) OR EXCLUDE ( EXACTKEYWORD , "Nonhuman" ) OR EXCLUDE ( EXACTKEYWORD , "Animal" ) OR EXCLUDE ( EXACTKEYWORD , "Animals" ) AND ( LIMIT-TO ( LANGUAGE , "English" ) ) |

| **Supplementary Table 2.** Minimally adjusted associations between menopausal hormone therapy use and liver cancer risk in the Million Women Study and UK Biobank | | | | | |
| --- | --- | --- | --- | --- | --- |
|  | **Million Women Study** | |  | **UK Biobank** | |
| **Menopausal hormone therapy use** | **Cases/N** | **HR (95% CI)** |  | **Cases/N** | **HR (95% CI)** |
| Never | 1,424/598,551 | 1 (ref) |  | 79/97,088 | 1 (ref) |
| Ever | 1315/XXXX | 1.01 (0.93-1.09) |  | 97/93,924 | 1.09 (0.81-1.48) |
| Former | 676/259,844 | 0.99 (XX-XX) |  | 81/78,999 | 1.04 (0.76-1.43) |
| Current | 73/380,720 | 0.85 (XX-XX) |  | 16/14,925 | 1.42 (0.83-2.44) |
| Duration of use |  |  |  |  |  |
| <5 years | 581/XXXX | 0.93 (0.XX-XX) |  | 49/52,262 | 1.06 (0.74-1.52) |
| 5-9 years | 429/XXX | 0.97 (0.XX-XX) |  | 20/20,185 | 1.02 (0.62-1.67) |
| 10+ years | 259/XXX | 1.01 (0.XX-XX) |  | 28/21,477 | 1.23 (0.79-1.92) |
| In the Million Women Study models were stratified by year of recruitment, year of birth, and adjusted for Townsend deprivation index and region.  In UK Biobank models were adjusted by region of recruitment, and deprivation and stratified by age at recruitment (<45, 45-49, 50-54, 55-59, 60-64, ≥65 years).  N represents the number of participants in each category. | | | | | |

| **Supplementary Table 3.** Characteristics of observational studies that assessed menopausal hormone therapy use in relation to liver cancer risk. | | | | | | | | |
| --- | --- | --- | --- | --- | --- | --- | --- | --- |
| **Case-control studies** | | | | | | | | |
| **First Author (year)** | **Country** | **Cases/controls, age at recruitment** | **Exposure** | **Exposure assessment** | **Outcome (number of cases/controls)** | **Control selection** | **Adjustment factors** | **OR (95% CI) ever vs never use and duration** |
| Yu, 1991 | USA | 25/58, mean case age 59.6 years, mean control age 58.8 years | Exposed case 10 (40%), exposed control 19(32.8%) | Interviewed via structured questionnaire in the home (2 by telephone) | Hepatocellular carcinoma (25) | Females from the surrounding neighborhood of the case | Matched on sex, year of birth (within 5 years) and race. Adjusted for use of oral contraceptives. | RR:  1.1 (0.3-3.6) |
| Yu, 2003 | Taiwan | 218 cases/565 high risk controls/164 spousal controls (729 total controls ), case mean age 58.5(8.9), women at high risk mean age 50.5(10.8), spouse mean age 49.3(8.7) | Exposed case 22 (10.1%), exposed controls 98(13.4%) | Interview via structured questionnaire | Hepatocellular carcinoma (218) | Female relatives who were free of diagnosed HCC | Adjusted for age at recruitment, history of diabetes, and status type and age of menopause | OR:  0.46 (0.27-0.79) |
| Fernandez, 2003 | Italy | 105/6,976, age range 45-79 | Hormone replacement therapy, case ever use 3 (2.8%), control never use approx. 530 (7.6%) | Interview conducted via trained interviewer | Liver cancer (105) | Hospital based controls admitted for acute conditions. | Adjusted for age, study center, year of interview, education, smoking, drinking, type/age at menopause, BMI, oral contraceptive use | OR:  0.2 (0.1-0.8) |
| Hassan, 2017 | USA | 234/282, cases <50 y/o: 41, cases ≥50 y/o: 193, control <50 y/o 42, controls GE 50 y/o 240 | Estrogen case ever 94 (40.2%), estrogen control ever 179 (63.5%), Progesterone case ever 38 (16.2%), progesterone control ever 63 (22.3%) | In person interview via structured questionnaire | Hepatocellular carcinoma (234) | Family members of cancer patients at MD Anderson not related to the incident case. | Adjusted for age, race, education level, marital status, hepatitis C virus, hepatitis B virus, alcohol drinking, cigarette smoking, history of diabetes, family history of cancer, obesity at age 20-40 years, hypothyroidism, oophorectomy and marital status. | OR:  0.42 (0.21-0.81) |
| **Cohort study** | | | | | | | | |
| **First Author (year)** | **Cohort name, country** | **Sample size, age at entry, follow up time** | **Exposure** | **Exposure Assessment** | **Outcome (number of cases)** | **Outcome assessment method** | **Adjustment Factors** | **HR (95% CI), ever vs never and duration** |
| Persson, 1996 | Sweden | 22, 597, Mean age 54.5, mean FUP 13.2 years | 100% (a cohort of MHT users) | Extracted from pharmacy records | Liver cancer (14) | National registration number linkage to Central Cancer Registry | Age matched, no adjustments | SIR: 0.7 (0.4-1.2) |
| Goodman, 1995 | Japan | 36133 total (does not give number of women or men specifically), mean age not described, mean follow up overall 8.61 years | Exposed case: 7, unexposed case: 69 | Self-administered survey | Liver cancer (76) | Incidence cases abstracted from medical records and supplemented with death records in Nagasaki and Hiroshima | Adjusted for city, age at time of bombings, attained age and radiation dose to the liver | RR: 1.29 (0.59-2.84) |
| McGlynn, 2015 | USA | 200 case/586,271 non-case, mean age not described, Mean follow up not described | Exposed case: 112 (56%), exposed non case: 335,084 (57.2%) | Various; data contributed from multiple studies | Hepatocellular carcinoma (200) | Documentation in a cancer registry or medical record | Adjusted for age, alcohol, BMI, diabetes, race, smoking, parent cohort study and education | HR: 1.35 (1.01-1.81) |
| McGlynn, 2016 | UK CPRD | 339/1,318, mean case age 68.1(13.8), mean control age 67.9(13.8) | Case 42 (12.4%), control 226 (17.2%) | Linkage to CPRD; data recorded by general practitioners | Liver cancer (339) | Linkage to CPRD | Adjusted for BMI, smoking, alcohol related disorders, HBV, HCV, diabetes, paracetamol use, rare metabolic disorders, aspirin, diabetes medications, bilateral oophorectomy, hysterectomy and statins | OR: 0.58 (0.37-0.9) |
| Simin, 2017 | Sweden | 290,186, mean age not described, median follow up 7 years | A cohort of those exposed MHT | Linkage to Swedish Prescribed Drug Registry | Liver cancer (94) | Linkage to the Cancer Registry and the Swedish Cause of Death Registry | Not described/not adjusted | SIR: 0.81 (0.65-0.99) |
| Petrick, 2020 | LCPP (USA) | 154 case/722,150 nonc-ase, mean age non case 57.7 (10.56), mean age case 61.9 (7.20), mean FUP 13.0 years | Exposed case 83 (53.9%), exposed non case 340,563 (47.2%) | Self-reported via questionnaire | Intrahepatic cholangiocarcinoma (154) | Linkage to cancer registries, medical/pathology record review | Age (continuous), alcohol (g/day: none, .1.08, >1.08.3.58, >3.58.13.54, >13.54), BMI (kg/m2: <25, 25.29.9, .30), diabetes (yes, no), race, smoking, parent cohort study, menopausal status, education | HR: 1.12 (0.80-1.56) |
| Tuo, 2022 | China | 35,555, mean case age 60.8 (14.7), mean non case age 50.2 (16.4), mean FUP 17.44 | Exposed case (7, 2.7%), exposed non case (2668, 3.5%) | Questionnaires by trained interviewers | Liver cancer (197) | Verification via home visits, medical reports/charts, by clinical and pathological experts | Age at entry, BMI, physical activity, calorie intake, education, family income, occupation, marital status, smoking, alcohol drinking, tea drinking, family history of liver cancer, medical history of hepatitis, cholelithiasis, type 2 diabetes, high bp | HR: 1.23 (1.17-1.30)^1^ |
| Chang, 2022 | Taiwan, China | 2,044 cohort (1022 MHT users, 1022 nonusers), mean age: 51.1(+/- 14.8), mean FUP among HRT users:7.47 years , mean FUP among nonusers:6.64 years | Exposed case: 61 (41%), exposed non case: 961(51%) | Linkage to National Health Research Database | Hepatocellular carcinoma (149) | Linkage to National Health Research Database | age, HCD, hyperlipidemia, diabetes, CAD, CKD, Sjogren syndrome and antiviral therapy status | HR: 0.49 (0.31-0.76) |
| Wang, 2022 | Taiwan | 6160, mean age 55.4 (+/-12.0) years , mean  FUP HRT cohort: 15.1(+/-4.1) years, mean FUP non-HRT cohort: 15.0(+/- 4.1) years | Exposed case: 19 (33.9%), exposed non case: 3061 (50.1%) | Linkage to Taiwan National Health Insurance Research Database (NHIRD) | Hepatocellular carcinoma (56) | Linkage to Taiwan National Health Insurance Research Database (NHIRD) | age, HCD, hyperlipidemia, DM, CKD, and CCI, | HR: 0.50 (0.29–0.87) |
| Lee, 2024 | South Korea | 29,466, mean age 55.5 (+/- 4.9), mean FUP not described | Exposed case: 67 (48.6% of cases), exposed non-case: 14,666 (50.0%) | Linkage to Korean Health Insurance Review and Assessment Service | Liver cancer (138) | Linkage to Korean Health Insurance Review and Assessment Service | Age at endometriosis diagnosis, year of endometriosis diagnosis, number of endometriosis surgeries and hysterectomy for benign disease | OR: 0.89 (0.63-1.24),  HR: 0.83 (0.59-1.17) |
| ^1^ Confidence intervals were deemed to be invalid due to the number of cases observed among women.  Abbreviations: BMI, body mass index; HCC, hepatocellular carcinoma; ICC, intrahepatic cholangiocarcinoma; ICD, international classification of disease. | | | | | | | | |

| **Supplementary Table 4.** Newcastle-Ottawa Quality grading of case-control studies included in systematic review and meta-analysis. | | | | | | | | | | |
| --- | --- | --- | --- | --- | --- | --- | --- | --- | --- | --- |
| First author, publication year | **Quality measure** | | | | | | | | **Total quality score (out of 9)** |  |
|  | Case definition adequate | Representative cases | Selection of controls appropriate | Definition of controls clear (No history of disease) | Appropriate adjustment for confounders | Ascertainment of exposure (MHT) | Same method of ascertainment of exposure between cases and controls | Non-response rate same for both cases and controls |  |  |
| Yu, 1991 | 1 | 1 | 1 | 0 | 1 | 0 | 1 | 0 | 5 |  |
| Yu, 2003 | 1 | 1 | 0 | 1 | 2 | 0 | 1 | 1 | 7 |  |
| Fernandez, 2003 | 1 | 1 | 0 | 0 | 1 | 0 | 1 | 1 | 5 |  |
| Hassan, 2017 | 1 | 0 | 0 | 1 | 2 | 0 | 1 | 1 | 6 |  |
|  |  |  |  |  |  |  |  |  |  |  |
| First author, publication year (cohort) | **Quality measure** | | | | | | | | | **Total Quality Score (out of 9)** |
|  | Representative study | Selection of non-exposed cohort | Ascertainment of exposure (MHT) | Outcome not present at baseline | Comparable exposure | Appropriate adjustment ^a^ | Record linkage used for outcome | Follow-up, >4 years | Adequacy of follow-up |  |
| Goodman, 1995 | 0 | 1 | 0 | 1 | 1 | 0 | 1 | 1 | 1 | 6 |
| Persson, 1996 | 0 | 0 | 1 | 0 | 0 | 0 | 1 | 1 | 1 | 4 |
| McGlynn, 2015 (LCPP) | 1 | 1 | 1 | 1 | 1 | 1 | 1 | 1 | 1 | 9 |
| McGlynn, 2016 | 1 | 1 | 1 | 1 | 1 | 1 | 1 | 1 | 1 | 9 |
| Simin, 2017 | 0 | 0 | 1 | 1 | 0 | 0 | 1 | 1 | 1 | 5 |
| Petrick, 2020 | 1 | 1 | 1 | 1 | 1 | 1 | 1 | 1 | 1 | 9 |
| Tuo, 2022 | 1 | 1 | 1 | 1 | 1 | 1 | 1 | 1 | 1 | 9 |
| Chang, 2022 | 0 | 1 | 0 | 1 | 1 | 0 | 1 | 1 | 1 | 6 |
| Wang, 2022 | 0 | 1 | 0 | 1 | 1 | 0 | 1 | 1 | 1 | 6 |
| Lee, 2024 | 0 | 1 | 1 | 1 | 1 | 0 | 1 | 0 | 1 | 6 |

^a^ Defined as adjustment for at least age, sex, smoking, alcohol intake, and a measure of socioeconomic status.

# Supplementary Figures

1,364,315 Million Women Study Sample

**44,431** diagnosed with cancer at baseline

1,319,884 participants

**47,285** had unknown/missing information on menopausal hormone therapy

1,272,599 participants

**58** lost to follow-up

1,272,541 females

**Supplementary Figure 1.** Flow chart of exclusion criteria applied to the Million Women Study sample.

503,317 UK Biobank Sample

**961** participants withdrew consent/ineligible

502,356 participants

**28,294** diagnosed with cancer at baseline

474,062 participants

**219,303** reported male sex

254,759 participants

**63,747** were not postmenopausal at baseline or had unknown menopausal hormone therapy use

191,012 females

**Supplementary Figure 2.** Flow chart of exclusion criteria applied to the UK Biobank sample.

**Supplementary Figure 3.** Subgroup and sensitivity analyses for ever versus never menopausal hormone therapy use and risk of liver cancer in Million Women Study and UK Biobank.

Never use of menopausal hormone therapy was the reference group.

Number of cases represent the total number of cases in the analyses (for both never and ever users of menopausal hormone therapy).

Million Women Study analyses were stratified by year of recruitment, year of birth, and adjusted for deprivation (Townsend deprivation index quintiles), region of recruitment, body mass index, height, physical activity, smoking status, alcohol consumption, education, diabetes status, menopausal status/years since menopause, menopausal hormone therapy use, and history of a hysterectomy.

UK Biobank analyses were stratified by age at recruitment, and adjusted for region of recruitment, deprivation, body mass index, height, physical activity, smoking status, alcohol consumption, coffee intake, ethnicity, education, diabetes status, menopausal status, menopausal hormone therapy use, history of a hysterectomy.

P-het represents the p-value for heterogeneity between subgroups obtained from χ^2^ for the difference between hazard ratios of liver cancer associated with ever versus never use of menopausal hormone therapy for the subgroups of interest.

Records identified through database search
(n=7,752)

• CINAHL (n = 94)

• Embase (n = 2,051)

• PubMed (n = 1,991)

• Scopus (n= 1,719)

• Web of Science (n = 1,897)

**Identification**

**Identification**

Records removed *before screening*
(n = 3,016)

Reason: Duplication

Reports assessed for eligibility
(n = 330)

Studies included in meta-analysis

(n=14)

Records screened
(n = 4,736)

Records excluded
(n = 4,406)

Reports sought for retrieval
(n = 330)

**Screening**

**Screening**

Reports not retrieved
(n = 0)

Reports excluded: (n = 316)

- Wrong study design (n=228)
- Systematic review (n=1)
- Did not assess menopausal hormone therapy (n= 43)
- Did not assess liver cancer as the outcome (n= 40)
- Updated analysis in same cohort/ case-control participants (n=3)
- Duplicate (n=1)

**Included**

**Supplementary Figure 4.** PRISMA flow diagram of the systematic review literature search of observational studies assessing menopausal hormone therapy use in relation to liver cancer risk.


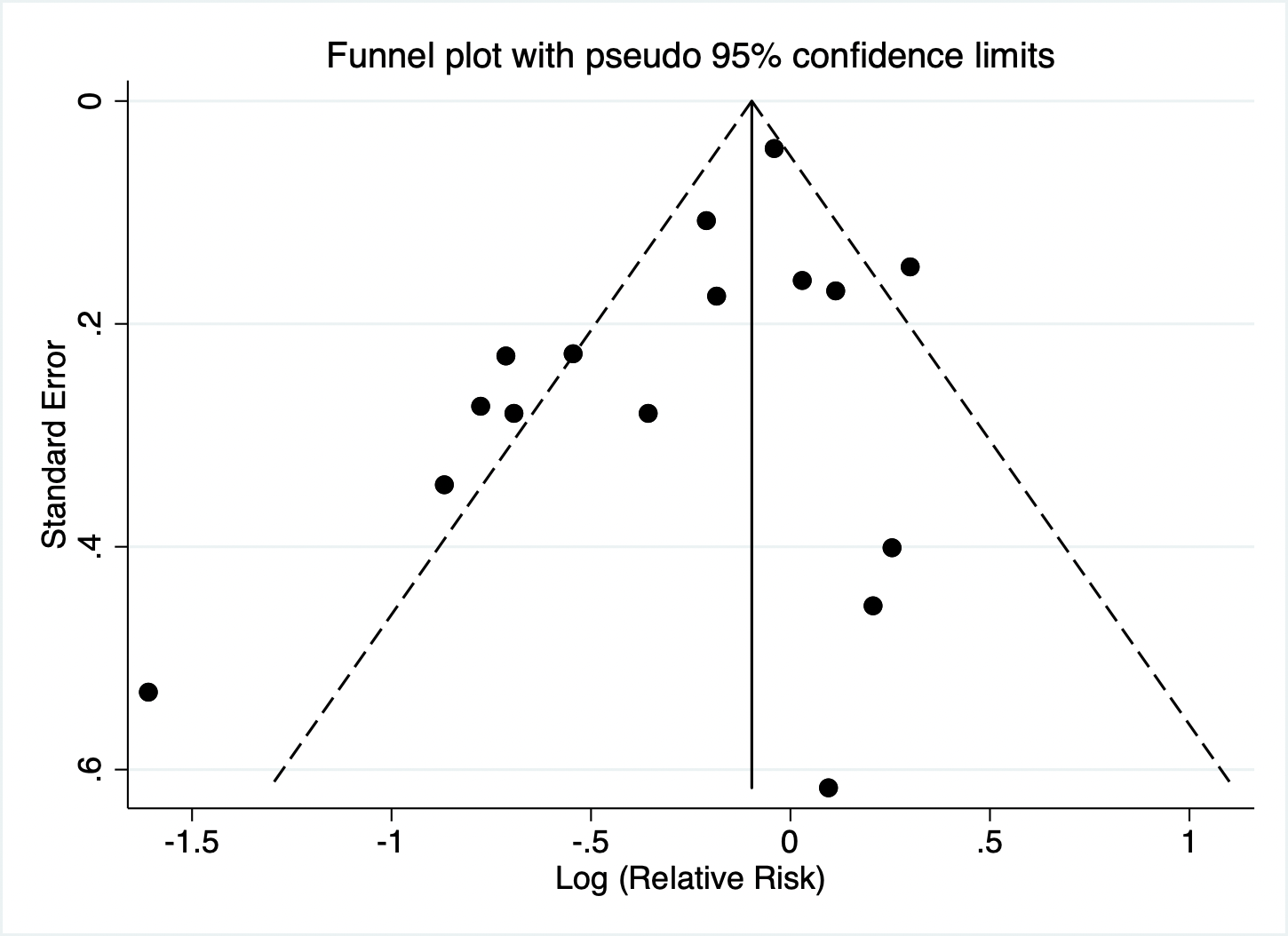


**Supplementary Figure 5.** Funnel plot with pseudo 95% confidence intervals for ever versus never use of menopausal hormone therapy and liver cancer risk.

**Supplementary Figure 6.** Meta-analysis of all observational studies which assessed ever versus never consumers of menopausal hormone therapy in relation to the risk of liver cancer restricted to high quality studies.

Values for number of cases listed here represent the number of liver cancer cases/controls for case-control studies and the number of liver cancer cases observed and total number of participants for prospective cohort studies

Abbreviations: CRPD, Clinical Practice Research Datalink; CI, confidence intervals; HCC, hepatocellular carcinoma; ICC, intrahepatic cholangiocarcinoma; LCPP, Liver Cancer Pooling Project; MWS, Million Women Study; RR, relative risk; UK, United Kingdom; USA, United States of America.


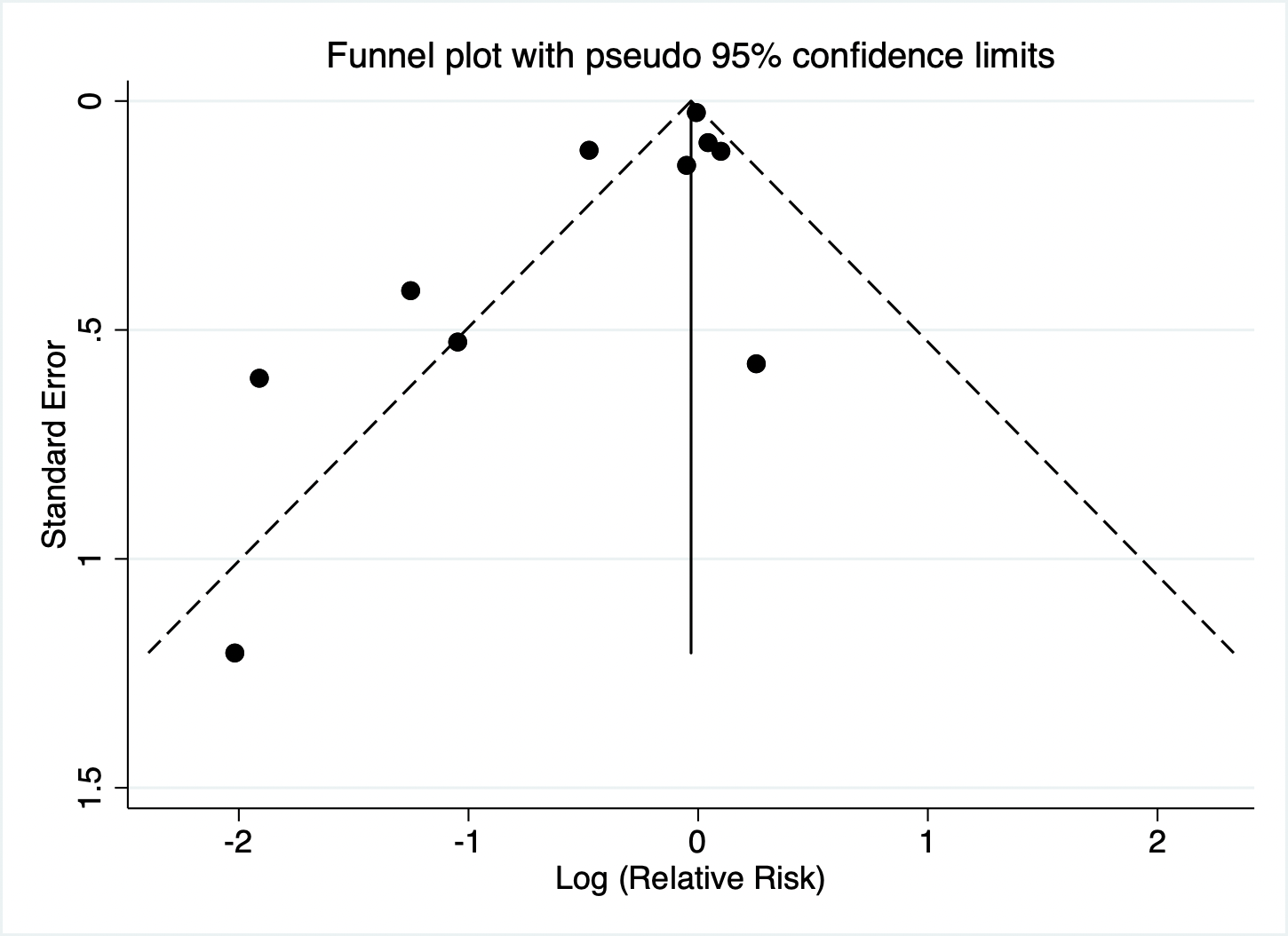


**Supplementary Figure 7.** Funnel plot with pseudo 95% confidence intervals for duration of use of menopausal hormone therapy and liver cancer risk.

**Supplementary Figure 8.** Meta-analysis of all observational studies which assessed long-term consumers of menopausal hormone therapy in relation to the risk of A) hepatocellular carcinoma; and B) intrahepatic cholangiocarcinoma.

Abbreviations: CI, confidence intervals; HCC, hepatocellular carcinoma; ICC, intrahepatic cholangiocarcinoma; RR, relative risk.

**Supplementary Figure 9.** Meta-analysis of highest duration of menopausal hormone therapy use category compared to never users and A) risk of liver cancer; B) risk of hepatocellular carcinoma; and C) intrahepatic cholangiocarcinoma.

Meta-analysis estimates are derived from fixed effects models.

Abbreviations: CI, confidence intervals; MHT, menopausal hormone therapy; MWS, Million Women Study; RR, relative risk.

**Supplementary Figure 10.** Meta-analysis of all observational studies which assessed different formulations of menopausal hormone therapy in relation to the risk of A) all liver cancer; B) hepatocellular carcinoma; and C) intrahepatic cholangiocarcinoma.

Abbreviations: CI, confidence intervals; HCC, hepatocellular carcinoma; ICC, intrahepatic cholangiocarcinoma; RR, relative risk.
