# Supplementary material for: Oral contraceptive use and risk of liver cancer: a population-based study, systematic review, and meta-analysis
Source: Lancet Oncol. 2025 Aug;26(8):1031–42. doi: 10.1016/S1470-2045(25)00222-0 (PMC12303860; doi:10.1016/S1470-2045(25)00222-0)
Supplement: Supplementary file 1 [file mmc1.pdf]

THE LANCET
Oncology

Supplementary appendix

This appendix formed part of the original submission and has been peer reviewed. We post it as supplied by the authors.

Supplement to: Watling CZ, Sweetland S, Wojt A, et al. Oral contraceptive use and risk of liver cancer: a population-based study, systematic review, and meta-analysis. *Lancet Oncol* 2025; published online July 2. https://doi.org/10.1016/S1470-2045(25)00222-0.

Oral contraceptive use and risk of liver cancer: a population-based study, systematic review, and meta-analysis

Table of Contents

Covariate classification ... 2
Systematic Review and Meta-analysis ... 8
Supplementary Tables ... 11
Supplementary Figures ... 25
PROSPERO protocol ... 33

Covariate classification

In the Million Women Study, participants with missing information for each covariate were assigned into a missing indicator category. For UK Biobank, we utilised different methods for missing variables due to the smaller number of cases observed in this cohort. If a variable was missing for >5% of the study population, a missing indicator category was utilised. If a continuous variable was missing for <5% of the study population, the median value was used to replace the missing variable. If a categorical variable was missing for <5% of the study population, participants were randomly assigned to categories based on the distribution of the variable in participants that provided information on the variable.

Region

In the Million Women Study, participants were grouped into ten broad regions of residence based on the 10 regional cancer registries (9 regions in England – Oxford, East Anglia, South West, Thames, West Midlands, North Yorkshire, Trent, North West (Mersey), North West (Manchester / Lancashire) – and Scotland).

In UK Biobank, participants were grouped into ten broad regions based on the centre at which they were recruited: London (assessment centres: St Bartholomew's Hospital, Hounslow, Croydon), Wales (assessment centres: Swansea, Wrexham, Cardiff), North-West England (assessment centres: Stockport, Manchester, Liverpool, Bury), North-East England (assessment centres: Newcastle, Middlesbrough), Yorkshire (assessment centres: Leeds, Sheffield), West Midlands (assessment centres: Stoke, Birmingham), East Midlands (assessment centre: Nottingham), South-East England (assessment centres: Oxford, Reading), South-West England (assessment centre: Bristol), Scotland (assessment centres: Glasgow, Edinburgh).

Body mass index

Body mass index (BMI) was determined in the Million Women Study from self-reported height and weight at recruitment. From this, BMI was calculated, and women were categorised into the

following categories <20, 20–<25, 25–<30, and 30+ kg/m^2 and an unknown category if information was missing/unknown (~5%). In UK Biobank, participants had their weight and height measured by trained professionals, and from this, were categorised into the following categories: <25, 25.0-29.99, 30.0-34.99, 35-39.99, ≥40 kg/m^2. Women with missing information (0.51%) were assigned the median value for BMI (26.9 kg/m^2) and categorized into the respective category.

Height

In the Million Women Study, height was self-reported at recruitment and women were categorised into the following categories:<160, 160 to ≤164 and 165+ cm or unknown (1.5% of women). In UK Biobank, height was modelled as a continuous variable per 1 cm increase. Participants with missing information in UK Biobank (0.5%) were assigned the median value 162 cm.

Physical activity

In the Million Women Study, women were categorised based on self-reported strenuous activity into the following categories: <once per week, 1-3 times per week and 4+ times per week, with those with missing information assigned to an unknown category (3.6%). In UK Biobank, physical activity was determined from questions on the touchscreen questionnaire which asked about walking, moderate physical activity, and vigorous physical activity. These were used to estimate excess metabolic equivalent (MET)-hours/week of physical activity during work and leisure time. For each of the three activity categories (walking, moderate physical activity and vigorous physical activity), participants were asked how many days in a typical week they did each of the activities for 10 minutes or more. For each category, participants who entered one or more days were then asked how many minutes they spent doing those activities on a typical day. For each activity category, the number of reported days was multiplied by the number of reported minutes on a typical day to generate duration of activity in minutes per week. Activity on a typical day of 1260 min per week (equivalent to an average of 3 hours per day) were truncated at 1260. Total MET values for each category from the International Physical Activity Questionnaire short form were: 3.3 for walking, 4.0 for moderate physical activity and 8.0 for vigorous physical activity. Excess MET values were therefore 2.3 for walking, 3.0 for moderate

physical activity and 7.0 for vigorous physical activity. Excess MET-hours per week were calculated by multiplying the excess MET value for each activity by the duration of activity in hours per week. Participants with missing information (5.4%) were categorized into a 'missing' category.

Smoking status

In the Million Women Study, smoking status was categorised as never, former, current <15 cigarettes a day and current 15+ cigarettes a day based on responses from the recruitment questionnaire. If smoking status could not be determined, participants were categorised into a missing category (5.7% of participants). In UK Biobank, smoking status was categorised as never, former, or current smoker and was determined from questions from the recruitment questionnaire. Participants were asked "Do you smoke tobacco now?" and "in the past, how often have you smoked tobacco?" to determine their smoking status. Participants with missing information on smoking status (0.6%) were randomly assigned into one of the three categories.

Alcohol consumption

In the Million Women Study, alcohol intake was determined based on the number of drinks women had per week. From this, women were categorised as non-drinkers (< 1per week), 1-2.5 drinks per week, 3-6.5 drinks per week, 7-14.5 drinks per week, and 15+ drinks per week or unknown/missing (0.7% of participants). In UK Biobank, women were asked on the baseline questionnaire at recruitment how often they drank alcohol with the possible responses being: "daily or almost daily", "three or four times a week", "once or twice a week", "one to three times a month", "special occasions only", "never", or "prefer not to answer". Participants were then asked about their weekly or monthly intake of pints of beer, glasses of red wine, glasses of white wine/champagne, glasses of fortified wine, measures of spirits/liqueurs and glasses of other alcohol. A pint of beer was assumed to contain 20 grams of alcohol, and all other drinks contained 10 grams of alcohol. We then summed their total weekly or monthly consumption of alcohol accordingly. If the participant reported "do not know" or "prefer not to answer" to one of these questions on weekly or monthly consumption, they were coded as missing, except for "other alcohol", in which case we assigned them 0 grams from other alcohol. We used participants reported weekly consumption of alcohol. If this was unknown, due to the participant

reporting they only drank alcohol one to three times months or on special occasions, we used monthly consumption, if available. To get an estimated daily total, we divided weekly consumption by 7 (or monthly consumption by 30.4375). Alcohol consumption was categorised as <1 g/day, 1-9.99 g/day, 10-19.99 g/day, and ≥20 g/day, never or unknown. For participants who had unknown grams/day of alcohol but who reported consuming alcohol intake on “special occasions”, we assigned them to the category of “<1 g/day”. A total of 1.61% of participants had missing information and we replaced their missing value with mean intake (18.1 g/day) and categorized these participants accordingly.

Deprivation Index

In both the Million Women Study and UK Biobank, the Townsend deprivation index was based on the preceding national census output areas. Each participant was assigned a score in correspondence to the output area in which their postcode was located. From this, participants were categorised into quintiles from most deprived to least deprived. Participants with missing information in UK Biobank (0.1%) were randomly assigned into one of the 5 quintiles whereas in the Million Women Study participants were categorised into a missing category (0.7% of participants).

Education

In the Million Women Study, participants were categorized into the following categories: no educational qualifications, technical educational qualifications, secondary educational qualifications and tertiary educational qualifications based on answers to questions on the recruitment questionnaire. In UK Biobank, participants were asked ‘Which of the following qualification do you have?’ being able to select more than one. Possible answers were: College or University degree; A levels/AS levels or equivalent; O levels/GCSEs or equivalent; CSEs or equivalent; NVQ or HND or HNC or equivalent; Other professional qualifications example: nursing, teaching; None of the above; Prefer not to answer. We grouped participants into the following categories, based on their highest reported level of education: College or University degree, vocational qualifications (other professional qualifications/NVQ or HND or HNC), optional national exams at ages 17 to 18 years (A levels/AS levels), national exams at age 16

years (O levels/GCSEs/CSEs), none of the above, prefer not to answer, and missing (unknown/missing)).

Coffee intake

In the Million Women Study, coffee intake was not asked on the baseline questionnaire and therefore was not adjusted for. In UK Biobank, women were asked at recruitment how many cups of regular coffee they drink each day. From their responses we categorised participants as none, 1 cup/day, 2 cups/day, 3-4 cups/day, and 5+ cups a day. Around 7.56% of participants did not answer this question so they were assigned into a missing category variable.

Ethnicity

In the Million Women Study, women were not asked about their ethnicity on the baseline questionnaire and therefore this was not adjusted for. Ethnicity was asked in follow-up questionnaires and the large majority of women reported that they were white (96%), therefore adjustment for ethnicity is unlikely to influence these results. In UK Biobank, ethnicity of participants was determined from questions in the touchscreen questionnaire "What is your ethnic group?". Options included: White, Mixed race or other, Asian or Asian British, Black or Black British, Chinese, and other ethnic group. From this, women were categorised into two groups, white or non-white participants. Participants with missing information (0.5%) were randomly assigned based on distribution of participants to categories.

Diabetes status

In the Million Women Study, living with diabetes was established based on participants' self-reported diagnosis of, or treatment for, diabetes at recruitment (in response to the questions: "Have you ever been diagnosed with diabetes" or "Are you now being treated for diabetes"). From this, participants were categorised into yes, no, or unknown (0.09%). In UK Biobank, participants' diabetes status was determined using multiple questions from recruitment. First, from the question "Has a doctor ever told you that you have diabetes?" participants were classified as "yes", "no" or "unknown" based on their response. As well, participants who reported using metformin or insulin at recruitment were considered to have diabetes and included in the "yes" category. Finally, if a participant had a measured glycated hemoglobin (HbA1c) of ≥

48 mmol/mol at recruitment, they were defined as having diabetes and included in the "yes" category. Participants with missing or unknown information on diabetes status (<0.5%) were randomly assigned to categories.

Menopausal status

We determined menopausal status in the Million Women Study based on questions asked on the baseline questionnaire and from this, women were categorised as pre/perimenopausal, postmenopausal with time since menopause at recruitment specified as <5 years, 5-9 years, or 10+ years at study entry, postmenopausal but time since unknown, or as having unknown menopausal status (15.6% of participants). In the UK Biobank, women were categorised into premenopausal, postmenopausal: based on time since menopause <5 years, 5-9 years, or 10+ years at study entry, or unknown. Menopausal status was first determined by multiple questions asked on the baseline questionnaire and females were defined as being postmenopausal if they:

- Answered "yes" to having gone through menopause
- Answered "not sure" or did not answer if they had gone through menopause and:
    - were ≥55 years of age, or
    - had a bilateral oophorectomy

Women were defined as being pre-menopausal if they:

- Answered 'no' to the question regarding having gone through menopause, or
- Reported they were 'not sure' or did not respond to if they had gone through menopause and:
    - Were <50 years of age, did not have a bilateral oophorectomy/hysterectomy, and reported they were not using menopausal hormone therapy.
    - Were <50 years of age, reported they were menstruating today, and did not have a bilateral oophorectomy/hysterectomy.

All other women that did not fit into these two categories were categorised as unknown.

Menopausal hormone therapy use

In the Million Women Study, use of menopausal hormone therapy (MHT) was reported at recruitment. From this, participants were categorised as never, past or current users of MHT, or as having unknown MHT use if information was missing (~1% of participants). In UK Biobank, participants were categorised as "current", "former" and "never" MHT users based on the

questions asked about MHT use in the touchscreen questionnaire at recruitment. Women were asked “Have you ever used hormone replacement therapy?” and if they answered yes: “How old were you when you last used MHT?”. Women were asked to enter their age when they last used MHT or could choose “Still taking MHT”, or they could select “prefer not to answer” or “do not know”. Participants with missing information (<0.1%) were randomly assigned into categories due to low percentage of missingness.

Hysterectomy

In the Million Women Study, having a hysterectomy was determined from self-report and from hospital admission records (with OPCS4 codes Q07 and Q08) before study recruitment (for the small proportion of women with hospital records before recruitment). In UK Biobank, having a hysterectomy was only determined from self-report. From this, women were categorised as “no”, “yes”, or” unknown”. In total in UK Biobank, 11.5% of participants were listed as unknown/missing and a missing category was utilised, whereas 0.6% of participants in the Million Women Study had unknown hysterectomy status.

Systematic Review and Meta-analysis

The systematic review and meta-analysis was registered with PROSPERO (PROSPERO Number: CRD42024552518; full protocol available in Appendix on pp 33-35).

Literature search

A research librarian at the National Cancer Institute (GB) carried out the search of the literature for published articles that assessed the association of exogenous hormone use and liver cancer risk from all observational studies. Searching was conducted from database inception to June 28th, 2024. The following databases were searched: Embase, PubMed/MEDLINE, CINAHL, Scopus, and Web of Science for observational studies examining the exogenous hormone use with liver cancer risk. Supplementary Table 1 presents the search strategies employed across databases. Once titles and abstracts were obtained, two independent researchers (CZW, AW), using Covidence web-based systematic review screening software (https://app.covidence.org/), independently screened records for eligibility and selected abstracts to be fully reviewed. Once

abstracts were obtained, full texts of articles were searched, and reviewers made decisions whether to include or exclude. Any discrepancies were discussed between the reviewers until a consensus was reached. If no consensus was reached, a third reviewer (KAM) made the final decision.

Study selection

The inclusion criteria for the studies were: 1) observational studies, 2) presented in English, 3) studies that assessed the association between oral contraceptives with liver cancer (all or any subtype of liver cancer), and 4) studies that reported hazard ratios, odds ratios, or relative risks (RR) with 95% confidence intervals or presented the cases/controls within each category in which we could estimate odds ratios and 95% confidence intervals. We excluded any studies that were cross-sectional, did not assess liver cancer risk, did not look at oral contraceptive use (e.g., intrauterine contraceptives), or the main text was not available in English.

Data extraction

From the identified studies that met the eligibility criteria, one author extracted the study information including information on RR and 95% CI within each category of oral contraceptive use (ever vs. never) as well as for categories of duration of oral contraceptive use with risk of liver cancer. We also extracted the last name of author, publication year, country, cohort name (if applicable), sample size, mean age at entry, mean/median follow-up time (for cohort studies), total number of cases, number of cases and participants within each category, outcome ascertainment, and covariates adjusted for.

Risk of bias

The Newcastle-Ottawa Quality Assessment Scale for case-control studies and prospective studies was used to assess quality of studies. Specifically, two authors (CZW, AW) assessed the risk of bias using a slightly adapted Newcastle-Ottawa Quality Assessment Scale separately for the case-control studies and prospective studies. Case-control studies that scored ≥6 out of 8 were considered of high quality, whereas prospective studies that scored at least 8 out of 9 were considered high quality.[2]

Oral contraceptive duration of use meta-analysis

For articles that assessed associations between oral contraceptive use duration and liver cancer risk, we obtained the duration of use categories used, number of cases/controls/participants in each category, and relative risks, to determine associations per 5 years of use in a dose-response meta-analysis. If the number of controls was not presented, we replaced this with the same number of cases. If the number of participants from cohort studies was not presented, we divided the number of people in the cohort by the number of categories. For each of the categories presented with a range, we replaced this with the middle value. For example, if a study presented categories of oral contraceptive use by never, <1 years, 1 to <3 years, 3 to <6 years, 6 to <8 years, and 8+ years, we replaced these categories to 0 years, 0.5 years, 2 years, 4.5 years, 7 years, and 11 years, respectively. For the highest category, duration of use was approximated by adding 3 years to the cut off of the category in order to approximate the median duration. This was based on the duration of use in Million Women Study observed among the highest category (13 years for women in the 10+ years of use category). Using generalised least squares, we estimated associations per 5 years of oral contraceptive use in each of these studies. Studies that only included two categories for duration of use that was less than 5 years (e.g., <24 months, and ≥24 months) were not included in the generalised least squares trend meta-analysis, as they did not provide sufficient data to estimate a linear dose-response per 5 years of use. For Million Women Study and UK Biobank, we modelled associations in multivariable models per 5 years of oral contraceptive use and used the presented hazard ratio and 95% confidence intervals. A fixed effects meta-analysis was used to combine these estimates to determine associations per 5 years of oral contraceptive use. For the highest category meta-analysis, we included the highest category of duration of oral contraceptive use in each study. We did not include studies where the highest duration of oral contraceptive use was <5 years.

Supplementary Tables

Supplementary Table 1. Search strategy for systematic literature review.

Database: PubMed/MEDLINE
Platform: National Library of Medicine

	Concept	Search Strategy
#1	Exogenous Hormone	"Hormone Replacement Therapy"[Mesh] OR "hormon* replacement therap*"[Title/Abstract] OR "menopausal hormone therapy"[Title/Abstract:~4] OR "menopausal hormone therapies"[Title/Abstract:~4] OR "menopausal hormonal treatment"[Title/Abstract:~4] OR "menopausal hormonal treatments"[Title/Abstract:~4] OR "hormone substitution"[Title/Abstract] OR (("Postmenopause"[Mesh] OR "postmenopaus*"[Title/Abstract] OR "post-menopaus*"[Title/Abstract]) AND ("hormon* treatment*"[Title/Abstract] OR "hormon* therap*"[Title/Abstract])) OR "estrogen replacement"[Title/Abstract:~6] OR "estrogen therapy"[Title/Abstract:~6] OR "estrogen therapies"[Title/Abstract:~6] OR "oestrogen replacement"[Title/Abstract:~6] OR "oestrogen therapy"[Title/Abstract:~6] OR "oestrogen therapies"[Title/Abstract:~6] OR "oestrogen treatment"[Title/Abstract:~6] OR "estrogen treatment"[Title/Abstract:~6] OR "estrogen treatments"[Title/Abstract:~6] OR "exogenous hormon*"[Title/Abstract] OR "Contraceptives, Oral"[Mesh] OR "oral contracept*"[Title/Abstract] OR "Estradiol"[Mesh] OR "estradiol*"[Title/Abstract] OR "Spironolactone"[Mesh] OR "spironolactone*"[Title/Abstract] OR "Cyproterone Acetate"[Mesh] OR "cyproterone acetate*"[Title/Abstract] OR "Azasteroids"[Mesh] OR "azasteroid*"[Title/Abstract] OR "finasteride*"[Title/Abstract] OR "dutasteride*"[Title/Abstract] OR "Leuprolide"[Mesh] OR "leuprolide*"[Title/Abstract] OR "Goserelin"[Mesh] OR "goserelin*"[Title/Abstract] OR "histrelin" [Supplementary Concept] OR "histrelin*"[Title/Abstract] OR "Progesterone"[Mesh] OR "progesterone*"[Title/Abstract] OR "Progestins"[Mesh] OR "progestin*"[Title/Abstract]
#2	Liver Cancer	"Liver Neoplasms"[Mesh] OR "liver cancer"[Title/Abstract:~4] OR "liver cancers"[Title/Abstract:~4] OR "liver tumo*"[Title/Abstract] OR "hepat* cancer*"[Title/Abstract] OR "hepat* neoplasm*"[Title/Abstract] OR "hepat* carcinoma*"[Title/Abstract] OR "hepat* malignan*"[Title/Abstract] OR "hepat* tumor*"[Title/Abstract] OR "hepat* tumour*"[Title/Abstract] OR "hepat* adenoma*"[Title/Abstract] OR "intrahepatic cholangiocarcinoma*"[Title/Abstract] OR "intra-hepatic cholangiocarcinoma*"[Title/Abstract] OR (("Neoplasms"[Mesh] OR "neoplasm*"[Title/Abstract] OR "Carcinoma"[Mesh] OR "carcinoma*"[Title/Abstract] OR "malignan*"[Title/Abstract] OR "adenocarcinoma*"[Title/Abstract] OR "adenoma*"[Title/Abstract] OR "sarcoma*"[Title/Abstract] OR "metastasis"[Title/Abstract] OR "metastases"[Title/Abstract] OR "tumor"[Title/Abstract] OR "tumors"[Title/Abstract] OR "tumour*"[Title/Abstract]) AND ("Liver"[Mesh] OR "Liver Diseases"[Mesh Terms:noexp] OR "liver"[Title/Abstract]))
#3	Limits: Human & publication type	((#1 AND #2) NOT ("Animals"[Mesh] NOT ("Animals"[Mesh] AND "Humans"[Mesh]))) NOT ("editorial"[Publication Type] OR "comment"[Publication Type] OR "news"[Publication Type] OR "letter"[Publication Type] OR "Case Reports as Topic"[Mesh] OR "Case

		Reports"[Publication Type] OR "retracted publication"[Publication Type] OR "retraction of publication"[Publication Type] OR "retraction of publication"[Title/Abstract] OR "retraction notice"[Title] OR "retracted publication"[Title] OR "Congress"[Publication Type] OR "Consensus Development Conference"[Publication Type] OR "conference abstract*"[Title/Abstract] OR "conference proceeding*"[Title/Abstract] OR "conference paper*"[Title/Abstract] OR "proceeding*"[Title]) Filters: English

Database: Embase<br>Platform: Elsevier		
	Concept	Search Strategy
#1	Exogenous Hormone	'hormone substitution'/exp OR 'hormon* replacement therap*':ti,ab,kw OR (('menopausal' NEAR/4 'hormone therap*'):ti,ab,kw) OR (('menopausal' NEAR/4 'hormonal treatment*'):ti,ab,kw) OR 'hormone substitution':ti,ab,kw OR (('postmenopause'/exp OR 'postmenopaus*':ti,ab,kw OR 'post-menopaus*':ti,ab,kw) AND ('hormon* treatment*':ti,ab,kw OR 'hormon* therap*':ti,ab,kw)) OR ((estrogen NEAR/6 replacement):ti,ab,kw) OR ((estrogen NEAR/6 therap*):ti,ab,kw) OR ((oestrogen NEAR/6 replacement*):ti,ab,kw) OR ((oestrogen NEAR/6 therap*):ti,ab,kw) OR ((oestrogen NEAR/6 treatment*):ti,ab,kw) OR ((estrogen NEAR/6 treatment*):ti,ab,kw) OR 'exogenous hormon*':ti,ab,kw OR 'oral contraceptive agent'/exp OR 'oral contracept*':ti,ab,kw OR 'estradiol'/exp OR 'estradiol*':ti,ab,kw OR 'spironolactone'/exp OR 'spironolactone*':ti,ab,kw OR 'cyproterone acetate'/exp OR 'cyproterone acetate*':ti,ab,kw OR 'azasteroid'/exp OR 'azasteroid*':ti,ab,kw OR 'finasteride*':ti,ab,kw OR 'dutasteride*':ti,ab,kw OR 'leuprorelin'/exp OR 'leuprolide*':ti,ab,kw OR 'goserelin'/exp OR 'goserelin*':ti,ab,kw OR 'histrelin*':ti,ab,kw OR 'progesterone'/exp OR 'progesterone*':ti,ab,kw OR 'progestin*':ti,ab,kw
#2	Liver Cancer	'liver tumor'/exp OR 'liver tumo*':ti,ab,kw OR ((liver NEAR/4 cancer*):ti,ab,kw) OR 'hepat* cancer*':ti,ab,kw OR 'hepat* neoplasm*':ti,ab,kw OR 'hepat* carcinoma*':ti,ab,kw OR 'hepat* malignan*':ti,ab,kw OR 'hepat* tumor*':ti,ab,kw OR 'hepat* tumour*':ti,ab,kw OR 'hepat* adenoma*':ti,ab,kw OR 'intrahepatic cholangiocarcinoma*':ti,ab,kw OR 'intra-hepatic cholangiocarcinoma*':ti,ab,kw OR (('neoplasm'/exp OR 'neoplasm*':ti,ab,kw OR 'carcinoma'/exp OR 'carcinoma*':ti,ab,kw OR 'malignan*':ti,ab,kw OR 'adenocarcinoma*':ti,ab,kw OR 'adenoma*':ti,ab,kw OR 'sarcoma*':ti,ab,kw OR 'metastasis':ti,ab,kw OR 'metastases':ti,ab,kw OR 'tumor':ti,ab,kw OR 'tumors':ti,ab,kw OR 'tumour*':ti,ab,kw) AND ('liver'/exp/mj OR 'liver disease'/exp/mj OR 'liver':ti,ab,kw))
#3	Limits: Human & publication type	#1 AND #2 AND ([article]/lim OR [article in press]/lim) AND [english]/lim NOT ([animals]/lim NOT ([animals]/lim AND [humans]/lim)) NOT ([conference abstract]/lim OR [conference paper]/lim OR [conference review]/lim OR 'conference paper'/exp OR 'conference abstract*':ab,ti OR 'conference proceeding*':ab,ti OR 'proceeding*':ti OR [editorial]/lim OR 'editorial'/exp OR 'retraction notice'/exp OR 'retraction'/exp OR 'retraction of publication':ab,ti OR 'retraction notice':ti OR 'retracted publication':ab,ti OR [letter]/lim OR [note]/lim OR 'case report'/exp OR 'case report':ti)

Database: CINAHL Plus<br>Platform: EBSCOhost		
	Concept	Search Strategy
#1	Exogenous Hormone	((MH "Hormone Replacement Therapy") OR (MH "Contraceptives, Oral") OR (MH "Estradiol") OR (MH "Spironolactone") OR (MH "Leuprolide") OR (MH "Goserelin") OR (MH "Progesterone")) OR TI ("hormon* replacement therap*" OR (menopausal N4 "hormone therap*") OR (menopausal N4 "hormonal treatment*") OR "hormone substitution" OR (estrogen N6 replacement*) OR (estrogen N6 therap*) OR (oestrogen N6 replacement*) OR (oestrogen therap*) OR (oestrogen N6 treatment*") OR (estrogen N6 treatment*) OR "exogenous hormon*" OR "oral contracept*" OR "estradiol*" OR "spironolactone*" OR "cyproterone acetate*" OR "azasteroid*" OR "finasteride*" OR "dutasteride*" OR "leuprolide*" OR "goserelin*" OR "histrelin*" OR "progesterone*" OR "progestin*" OR (((MH "Postmenopause") OR "postmenopaus*" OR "post-menopaus*") AND ("hormon* treatment*" OR "hormon* therap*"))) OR AB ("hormon* replacement therap*" OR (menopausal N4 "hormone therap*") OR (menopausal N4 "hormonal treatment*") OR "hormone substitution" OR (estrogen N6 replacement*) OR (estrogen N6 therap*) OR (oestrogen N6 replacement*) OR (oestrogen therap*) OR (oestrogen N6 treatment*") OR (estrogen N6 treatment*) OR "exogenous hormon*" OR "oral contracept*" OR "estradiol*" OR "spironolactone*" OR "cyproterone acetate*" OR "azasteroid*" OR "finasteride*" OR "dutasteride*" OR "leuprolide*" OR "goserelin*" OR "histrelin*" OR "progesterone*" OR "progestin*" OR (((MH "Postmenopause") OR "postmenopaus*" OR "post-menopaus*") AND ("hormon* treatment*" OR "hormon* therap*")))
#2	Liver Cancer	(MH "Liver Neoplasms+") OR TI ((liver N4 cancer*) OR "hepat* cancer*" OR "hepat* neoplasm*" OR "hepat* carcinoma*" OR "hepat* malignan*" OR "hepat* tumor*" OR "hepat* tumour*" OR "hepat* adenoma*" OR "intrahepatic cholangiocarcinoma*" OR "intra-hepatic cholangiocarcinoma*" OR (((MH "Neoplasms") OR (MH "Carcinoma") OR "neoplasm*" OR "carcinoma*" OR "malignan*" OR "adenocarcinoma*" OR "adenoma*" OR "sarcoma*" OR "metastasis" OR "metastases" OR "tumor" OR "tumors" OR "tumour*") AND ((MH "Liver") OR (MH "Liver Diseases")OR "liver"))) OR AB ((liver N4 cancer*) OR "hepat* cancer*" OR "hepat* neoplasm*" OR "hepat* carcinoma*" OR "hepat* malignan*" OR "hepat* tumor*" OR "hepat* tumour*" OR "hepat* adenoma*" OR "intrahepatic cholangiocarcinoma*" OR "intra-hepatic cholangiocarcinoma*" OR (((MH "Neoplasms") OR (MH "Carcinoma") OR "neoplasm*" OR "carcinoma*" OR "malignan*" OR "adenocarcinoma*" OR "adenoma*" OR "sarcoma*" OR "metastasis" OR "metastases" OR "tumor" OR "tumors" OR "tumour*") AND ((MH "Liver") OR (MH "Liver Diseases")OR "liver")))
#3	Limits: Human & publication type	S1 AND S2 NOT ((MH "Animals+") OR (MH "Animal Studies")) NOT ((MH "Retracted Publication") OR (MH "Retraction of Publication) OR (MH "Congresses and Conferences")OR (MH "Case Studies")) Limiters - Peer Reviewed; English Language; Expanders - Apply equivalent subjects; Search modes - Proximity

Database: Web of Science (Core Collection)<br>Platform: Clarivate Analytics		
	Concept	Search Strategy
#1	Exogenous Hormone	TS=("hormon* replacement therap*" OR (menopausal NEAR/4 "hormone therap*") OR (menopausal NEAR/4 "hormonal treatment*") OR "hormone substitution*" OR (("postmenopaus*" OR "post-menopaus*") AND ("hormon* treatment*" OR "hormon* therap*")) OR (estrogen NEAR/6 replacement*) OR (estrogen NEAR/6 therap*) OR (oestrogen NEAR/6 replacement*) OR (oestrogen NEAR/6 therap*) OR (oestrogen NEAR/6 treatment*) OR (estrogen NEAR/6 treatment*) OR "exogenous hormon*" OR "oral contracept*" OR "estradiol*" OR "spironolactone*" OR "cyproterone acetate*" OR "azasteroid*" OR "finasteride*" OR "dutasteride*" OR "leuprolide*" OR "goserelin*" OR "histrelin*" OR "progesterone*" OR "progestin*")
#2	Liver Cancer	TS=((liver NEAR/4 cancer*) OR "hepat* cancer*" OR "hepat* neoplasm*" OR "hepat* carcinoma*" OR "hepat* malignan*" OR "hepat* tumor*" OR "hepat* tumour*" OR "hepat* adenoma*" OR "intrahepatic cholangiocarcinoma*" OR "intra-hepatic cholangiocarcinoma*" OR ("liver" AND ("neoplasm*" OR "carcinoma*" OR "malignan*" OR "adenocarcinoma*" OR "adenoma*" OR "sarcoma*" OR "metastasis" OR "metastases" OR "tumor" OR "tumors" OR "tumour*")))
#3	Limits: Human & publication type	#2 AND #1 and Preprint Citation Index (Exclude – Database) and Animals (Exclude – MeSH Headings) and Humans (MeSH Headings) and Case Report or Abstract or Meeting or Letter or Editorial Material or Book or Reference Material or News (Exclude – Document Types) and English (Languages) and Web of Science Core Collection (Database)

**Scopus**		
	Concept	Search Strategy
#1	Exogenous Hormone	(TITLE-ABS-KEY ("hormon* replacement therap*" OR (menopausal W/4 "hormone therap*") OR (menopausal W/4 'hormonal AND treatment*") OR " hormone AND substitution* " OR ((" postmenopaus* " OR " post-menopaus* ") AND (" hormon* AND treatment* " OR " hormon* AND therap* ")) OR (estrogen W/6 replacement*) OR (estrogen W/6 therap*") OR (oestrogen W/6 replacement*) OR (oestrogen W/6 therap*) OR (oestrogen W/6 treatment*) OR (estrogen W/6 treatment*) OR "exogenous hormon*" OR "oral contracept*" OR "estradiol*" OR "spironolactone*" OR "cyproterone acetate*" OR "azasteroid*" OR "finasteride*" OR "dutasteride*" OR "leupro lide*" OR "goserelin*" OR "histrelin*" OR "progesterone*" OR "progestin*")
#2	Liver Cancer	TITLE-ABS-KEY ((liver W/4 cancer*) OR "hepat* cancer*" OR "hepat* neoplasm*" OR "hepat* carcinoma*" OR "hepat* malignan*" OR "hepat* tumor*" OR "hepat* tumour*" OR "hepat* adenoma*" OR "intrahepatic cholangiocarcinoma*" OR "intra-hepatic cholangiocarcinoma*" OR

		("liver" AND ("neoplasm*" OR "carcinoma*" OR "malignan*" OR "adenocarcinoma*" O R "adenoma*" OR "sarcoma*" OR "metastasis" OR "metastases" OR "tumor " OR "tumors" OR "tumour*"))) AND NOT TITLE-ABS-KEY ("editorial" OR "letter" OR "case report*" OR "retracted publication" OR "conference abstract*" OR "conference proceeding*" OR "conference paper*")
#3	Limits: Human & publication type	NOT TITLE-ABS-KEY ("editorial" OR "letter" OR "case report*" OR "retracted publication" OR "conference abstract*" OR "conference proceeding*" OR "conference paper*") AND NOT TITLE ("retracted publication" OR "retraction notice" OR proceeding*))
#4		(TITLE-ABS-KEY ("hormon* replacement therap*" OR (menopausal W/4 "hormone therap*") OR (menopausal W/4 'hormonal AND treatment* ") OR " hormone AND substitution* " OR ((" postmenopaus* " OR " post-menopaus* ") AND (" hormon* AND treatment* " OR " hormon* AND therap* ")) OR (estrogen W/6 replacement*) OR (estrogen W/6 therap*") OR (oestrogen W/6 replacement*) OR (oestrogen W/6 therap*) OR (oestrogen W/6 treatment*) OR (estrogen W/6 treatment*) OR "exogenous hormon*" OR "oral contracept*" OR "estradiol*" OR "spironolactone*" OR "cyproterone acetate*" OR "azasteroid*" OR "finasteride*" OR "dutasteride*" OR "leupro lide*" OR "goserelin*" OR "histrelin*" OR "progesterone*" OR "progestin*") AND TITLE-ABS-KEY ((liver W/4 cancer*) OR "hepat* cancer*" OR "hepat* neoplasm*" OR "hepat* carcinoma*" OR "hepat* malignan*" OR "hepat* tumor*" OR "hepat* tumour*" OR "hepat* adenoma*" OR "intrahepatic cholangiocarcinoma*" OR "intra-hepatic cholangiocarcinoma*" OR ("liver" AND ("neoplasm*" OR "carcinoma*" OR "malignan*" OR "adenocarcinoma*" O R "adenoma*" OR "sarcoma*" OR "metastasis" OR "metastases" OR "tumor " OR "tumors" OR "tumour*"))) AND NOT TITLE-ABS-KEY ("editorial" OR "letter" OR "case report*" OR "retracted publication" OR "conference abstract*" OR "conference proceeding*" OR "conference paper*") AND NOT TITLE ("retracted publication" OR "retraction notice" OR proceeding*)) AND (LIMIT-TO (EXACTKEYWORD , "Human") OR LIMIT-TO (EXACTKEYWORD , "Humans") OR EXCLUDE (EXACTKEYWORD , "Nonhuman") OR EXCLUDE (EXACTKEYWORD , "Animal") OR EXCLUDE (EXACTKEYWORD , "Animals") AND (LIMIT-TO (LANGUAGE , "English"))

Supplementary Table 2. Minimally adjusted associations between oral contraceptive use and liver cancer risk in the Million Women Study and UK Biobank

	Million Women Study		UK Biobank	
**Oral contraceptive use**	**Cases/N**	**HR (95% CI)**	**Cases/N**	**HR (95% CI)**
Never	1264/530,974	1 (reference)	43/47,290	1 (reference)
Ever	1501/774,050	1.01 (0.93-1.09)	148/206,118	1.08 (0.76-1.53)
<5 years	595/310,614	0.99 (0.90-1.10)	36/52,917	0.87 (0.56-1.37)
5-9 years	386/209,657	0.99 (0.88-1.11)	41/45,149	1.34 (0.86-2.08)
10+ years	444/221,634	1.04 (0.93-1.17)	54/87,512	1.01 (0.66-1.53)

In the Million Women Study models were stratified by year of recruitment, year of birth, and adjusted for Townsend deprivation index and region.

In UK Biobank models were adjusted by region of recruitment, and deprivation and stratified by age at recruitment (<45, 45-49, 50-54, 55-59, 60-64, ≥65 years).

N represents the number of participants in each category.

Supplementary Table 3. Characteristics of observational studies that assessed oral contraceptive use in relation to liver cancer risk.

Case-control studies								
**First Author (year)**	**Country**	**Cases/controls, age at recruitment**	**Exposure**	**Exposure assessment**	**Outcome, number of cases/controls**	**Case assessment**	**Adjustment factors**	**OR (95% CI) ever vs never use and duration**
Henderson (1983)	USA	11/22, mean age = 27.8 years (7.6)	Oral Contraceptive, 10 (91%) case, 13 (59%) control	Structured telephone interview; 3 cases, 3 spouses, 4 parents, 1 physician	Liver cancer (11)	Linkage to LA country cancer registry	-	6.92 (0.75-64.02)
Forman (1986)	UK	30/147, age range = 20-44 years	Oral contraceptives case ever 18 (60%), control ever 79 (54%)	General practitioner questionnaire	Liver cancer (30), HCC (19), Cholangiocarcinoma (11)	Draft death entries from the Office of Population Censuses and Surveys	Age at diagnosis, year of birth	Ever: 3.8 (1.0-14.6)
Neuberger (1986)	England	26/1333, mean age not described	Oral contraceptives [case 18 (69.2%), control (actual numbers not given)]	Interviews by different interviews, questionnaires NOT standardized	Hepatocellular carcinoma (26)	Histological confirmation of HCC patients	Age groups and calendar period (date of diagnosis/interview: 1976-79, 1980-84)	Ever: 1.0 (0.4-2.4)<br><br><4 years: 0.3 (0.1-1.1)<br><br>4-7 years: 0.9 (0.3-3.4)<br><br>8+ years: 4.4 (2.5-12.8)
Palmer (1989)	USA	9/45, mean age = 41.25 years (10.7)	Oral contraceptives [case 11(91.7%), control (33.3%)]	Standardized questionnaire administered by trained nurses	Liver cancer (Hepatocellular carcinoma (9), cholangiocarcinoma (2), undetermined (1)	Hospital diagnoses classified by pathologist viewing discharge summaries and pathology reports	Matched on geographic location of the hospital, date of interview, five-year age category	Ever: 14.5 (1.66-125.6)
Molina (1989)	Chile, China, Colombia, Israel, Kenya, Nigeria,	122/802, case mean age = 41.8 years	Oral contraceptives [case 25 (20.5%), control 216 (27.0%)]	Standardized interviews by trained personnel, questionnaires	Liver cancer (122) (Hepatocellular carcinoma (36), Cholangiocarcinoma(30),	Initial diagnosis by local pathologist, histology slides sent to a single reference pathologist for	Matched on age group (5-year groups), study center, year of study interview.	All liver cancer ever: 0.71 (0.4-1.2),<br><br>HCC ever:

	Philippines, Thailand			completed in local language then transcribed to English	clinically diagnosed but nonspecific subtype(56))	uniform classification	Adjusted for number of live births and occupation	0.60 (0.2-1.6),<br><br>ICC ever:<br>1.22 (0.5-3.1),
La Vecchia (1989)[1]	Italy	21/145,<br>median age = 50 years	Oral contraceptives [case 4 (19%), control 11(7.6%)]	Interview via structured questionnaire	Hepatocellular carcinoma (21)	Histologically or serologically confirmed primary liver cancer	Age in decades	≤5 year:<br>1.8 (0.4-9.2)<br><br>>5 years:<br>8.3 (1.4-48.7)
Vall Mayans (1990)	Catalonia, Spain	29/57,<br>mean age = 65.0 years (9.1)	Oral contraceptives [case ever 6 (20.7%), control ever 3(5.3%)]	Interview via structured questionnaire	Hepatocellular carcinoma (29)	Histological or cytological confirmed HCC	Not adjusted	Ever:<br>4.69 (1.08-20.41)
Kew (1990)	South Africa	46/92,<br>mean age = 39.0 years	Oral contraceptives [case 15 (32.6%, control 34 (37.0%)]	Interview	Hepatocellular carcinoma (46)	Histologically confirmed HCC	Matched sex, race, exact age, tribe, place of birth (rural or urban) and subsequent geographical movements	Ever:<br>1.9 (0.6-5.6)<br><br>4-8 years:<br>2.0 (0.1-33.1),<br><br>>8 years:<br>1.5 (0.3-7.2)
Yu (1991)	USA	25/58,<br>mean age for women not described	Oral contraceptives [(case ever: 13 (52%), control ever: 18 (31%)],	Interview via structured questionnaire, in person except for 2 over telephone	Hepatocellular carcinoma (25)	Histologically confirmed cases identified through the Los Angeles County Cancer Surveillance Program	Adjusted for duration of use of Premarin/other estrogens, matched on age (within five years), sex and race	Ever:3.0 (1.0-9.0),<br><br>13-60 months:<br>1.7 (0.3-9.1)<br><br>61+ months: 5.5 (1.2-24.8)
Hsing (1992)	National Mortality Followback Survey - USA	All liver cancer = 76; ICC = 22; Controls=629<br><br>Age range 25-49,	Oral Contraceptive [case ever: 39 (54.2%), control ever: 243 (44.3%)]	Questionnaires mailed to next of kin	Primary liver cancer (76), Intrahepatic cholangiocarcinoma (22)	Death certificates	Age at death, race, annual family income, use of alcohol, smoking	All liver cancer ever: 1.6 (0.9-2.6)<br><br>5-9 yrs: 2.0 (1.0-4.4), ≥10 years 2.0 (0.8-4.8)<br><br>ICC ever:<br>0.8 (0.3-2.7)

								5-9 years: 0.6 (0.1-5.4)<br>≥10 yrs 3.3 (0.7-15.9)
Khella (1992)	Egypt	62/62, mean age 45.9 (13.1)	Oral contraceptive case 8, control 3	Interview questionnaire	Hepatocellular carcinoma (Female cases only unknown, all cases HCC 62)	Not described	Not described	Ever:<br>2.91 (0.65- 14.7)
Tavani (1993)	Italy	82/368, median age 59	Oral contraceptives [9 (21.4%) cases, 21 (10.8%) controls]	Interview via structured questionnaire	Hepatocellular carcinoma (82)	Histological or serological confirmation	"Allowance was made for age, education and parity"	Ever: 2.6 (1.0-7.0)<br><br>>5 years:<br>3.9 (0.6-24.5)
Braga (1997)	Italy	85/377, all participant case median age 60), all participant control median age 56, female only age median not given	Oral contraceptives [case 9 (10.6%), control 25 (6.6%)]	Interview via structured questionnaire	Hepatocellular carcinoma (85)	Histological or serological confirmation	Age, area of residence, smoking status, education, total alcoholic beverage consumption, oral contraceptive use, history of hepatitis	Ever:<br>3.06 (1.2-8.1)
Heinemann (1997)	Multicentre International Liver Tumor Study - Germany, United Kingdom, France, Italy, Greece, Spain	cases/population controls/hospital controls 317/719/1060, case mean age 50.4(12.6), population control mean age 52.1(10.9), Hospital control mean age 47.7(12.6)	Oral contraceptives [case 148(50.5%), control 1986 (61%)]	Standardized interview via questionnaire	Hepatocellular carcinoma (317)	Imaging+ serological results, histological confirmation, expert group evaluation	Age, area , center, year of birth, year of diagnosis/interview, history of chronic diseases, occupation	Ever:<br>0.75(0.54-1.03),<br><br>3-5 years:<br>0.59 (0.33-1.06),<br><br>6+ yrs:<br>0.77 (0.54-1.12)
Yu (2003)	China	218/729,<br>Age cases = mean 58.5 years (8.9),<br><br>Age controls (women at high-risk) = mean age 50.5 years (10.8)	Oral contraceptives [case 20 (9.2%), control 110 (15.1%)]	Interview via structured questionnaire	Hepatocellular carcinoma (218)	Positive histology or serology + positive imaging results	Age at recruitment, history of diabetes, number of FTP	Ever:<br>0.75 (0.44-1.28),<br><br>≥24 months:<br>0.38 (0.13-1.09)
Kanazir (2010)	Serbia	13/26,	Oral contraceptives [case 1 (7.7%),	Interviewed by physician with	Hepatocellular carcinoma (13)	Histologically confirmed HCC	Matched on age	Ever: 0.6 (0.1-6.5)

		Age matched (no mean listed)	controls 3 (11.5%)]	standard questionnaire				
Hassan (2017)	USA	234/282<br><br>Age cases = 41 years<br><br>Age controls = 42 years	Oral contraceptives [case 146 (62.4%), control 208 (73.8%)]	In person interview	Hepatocellular carcinoma (234)	New patients with pathological or radiological evidence of HCC treated at MD Anderson	Age, race, education level, marital status, hepatitis C virus, hepatitis B virus, alcohol drinking, cigarette smoking, history of diabetes, family history of cancer, obesity at age 20-40 years, hypothyroidism, oophorectomy and marital status	Ever: 0.70 (0.42-1.18)<br><br>≤5 years: 0.41 (0.22-0.79)<br><br>6-10 years: 0.54 (0.26-1.15)<br><br>>10 years: 1.38 (0.74-2.60)
**Cohort study**								
**First Author (year)**	**Cohort name, country**	**Sample size, age at entry, follow up time**	**Exposure**	**Exposure Assessment**	**Outcome (number of cases)**	**Outcome assessment method**	**Adjustment Factors**	**HR (95% CI), ever vs never and duration**
Hannaford (2007)[1]	Royal College of General Practitioners' Oral Contraception Study - UK	N= 45,950<br><br>Mean age at recruitment = 29 years (6.6),<br><br>Follow-up range = recruitment 1968 to last year of follow-up= 2004	Oral Contraceptives 28,762(62.6%): ever, duration	General Practitioner report	Gallbladder or Liver (27) (ICD-8 155 & 156)	General practitioner report, linkage to NHS central registries	Age, parity, smoking, social status	Ever: 0.55 (0.26-1.17)<br><br>49-96 months: 0.42 (0.05-3.51),<br><br>≥ 97 months: 1.52 (0.38-6.07)
Rosenblatt (2009)	Shanghai Textile Industry Bureau - China	N= 258,956,<br><br>Follow-up = ~9.3 years	Oral Contraceptives 37,319 (14.4%) : ever, duration	Questionnaire	Liver (468)<br>(No code provided)	Comparison between Shanghai Textile Industry Bureau Tumor and Death Registry with Shanghai Cancer Registry and review of medical records	Age, parity, tubal ligation	Ever: 0.82 (0.60-1.13)<br><br>1-11 months: 0.86 (0.50-1.48)<br><br>12-59 months: 0.68 (0.41-1.14)<br><br>60-119 months:

								1.34 (0.73-2.45)<br><br>120+ months:<br>0.67 (0.32-1.44)
Vessey (2013)	Oxford Family Planning Association study - UK	N= 17,032,<br>Age range 25-39 years,<br><br>Follow-up time: recruitment = 1968-1974<br><br>Study end date = 2010	Oral Contraceptives 9,640 (56.6%), ever, duration	In clinic interviews, home or telephone interviews when necessary, or follow up form	Liver and gallbladder (28) (ICD-8 155 & 156)	Linkage to National Health Service Center Registries	Age	Ever: 1.1 (0.5-2.6)<br><br>≤48 months:<br>1.4 (0.4-4.2)<br><br>49-72 months:<br>1.0 (0.2-4.1)<br><br>73-96 months:<br>1.4 (0.3-5.0)<br><br>97+ months<br>0.8 (0.2-2.4)
McGlynn (2015)	Liver Cancer Pooling Project (11 cohorts) - USA	N=799,500<br><br>Age:<br><50 y/o 16%, 50-59 y/o 32.2%, 60-69 y/o 41.9%, 70 y/o 9.9%<br><br>Follow-up = varied between cohorts	Oral Contraceptives 319,967 (40%), ever, duration	Self-reported questionnaire	Hepatocellular Carcinoma (201)	Documentation in Cancer Registry report or medical report	Age, alcohol, BMI, diabetes, race, smoking, parent cohort study, menopausal status and education	Ever:<br>1.12 (0.82, 1.55)<br><br><1 year:<br>0.95 (0.47-1.91)<br><br>1 to <3 years:<br>1.00 (0.61-1.64)<br><br>3 to <6 years:<br>1.03 (0.47-2.25)<br><br>6 to <8 years:<br>1.27(0.73-2.22)<br>8+ years:<br>1.27 (0.75-2.15)
Iversen (2017)	Royal College of General Practitioners' Oral	N=46,022<br><br>Age at recruitment: 28.8 years (6.6)	Oral contraceptives use = 28,983 (63.0%), ever	General practitioner report	Liver and gallbladder cancer (66)<br>(ICD-8 155-156)	General practitioner report, linkage to NHS central registries	Age, parity, smoking, social status	Ever:<br>0.87 (0.45-1.69)

	Contraception Study - UK	Median follow-up= 40.7 years (IQR 6.1-44.6)						
Petrick (2020)	Liver Cancer Pooling Project (12 cohorts) - USA	N= 851,157,<br><br>Non-case mean age = 57.7 (10.56)<br><br>Case mean age = 61.9 (7.20)<br><br>Follow-up = varied between cohorts	Oral contraceptives 331,721 (39.0%), ever, duration	Self-reported questionnaire	Intrahepatic cholangiocarcinoma (154)	Linkage to state, provincial or country specific cancer registries	Age, alcohol, BMI, diabetes, race, smoking, parent cohort study, menopausal status, education	Ever:<br>1.06 (0.75-1.48)<br><br><1 years:<br>0.94 (0.52-1.69)<br><br>1-2.5 years:<br>1.09 (0.59-2.01)<br><br>2.5-6 years:<br>0.59 (0.24-1.49)<br><br>6-9 years:<br>1.00 (0.54-1.88)<br><br>9+ years:<br>1.71(1.01-2.89)
Tuo (2022)	Shanghai Women's Health Study - China	N= 72,807<br><br>Case mean age= 60.8 (14.7),<br>Non-case mean age=50.2 (16.4),<br><br>Mean follow-up= 17.44 years	Oral contraceptives 14,870 (20.4%), ever	In person interview and questionnaire	Liver cancer (258) (ICD-9 155)	Linkage to Shanghai Cancer Registry, Shanghai Vital Statistics Registry, and Shanghai Resident Registry	Age at entry, BMI, physical activity, caloric intake, education, family income, occupation, marital status, smoking, alcohol drinking, tea drinking, family history of liver cancer, medical history of hepatitis, cholelithiasis, diabetes, high blood pressure	Ever premenopausal<br>0.99 (0.97-1.02)[2]<br><br>Ever postmenopausal<br>1.06 (1.03-1.08)[2]

[1] This case/control study or cohort reported associations more than once, this study was only used for duration of oral contraceptive use estimates.
[2] Confidence intervals were deemed to be invalid due to the number of cases observed among women.
Abbreviations: BMI, body mass index; HCC, hepatocellular carcinoma; ICC, intrahepatic cholangiocarcinoma; ICD, international classification of disease.

Supplementary Table 4. Newcastle-Ottawa Quality grading of case-control studies included in systematic review and meta-analysis.

First author, publication year	Quality measure								**Total quality score (out of 8)**
	Case definition adequate	Representative cases	Selection of controls appropriate	Definition of controls clear (No history of disease)	Appropriate adjustment for confounders [a]	Ascertainment of exposure (oral contraceptive use)	Same method of ascertainment of exposure between cases and controls	Non-response rate same for both cases and controls	
Henderson, 1983	0	1	1	0	0	0	1	1	4
Forman, 1986	0	1	0	1	1	0	1	0	4
Neuberger, 1986	1	0	0	0	1	0	0	0	2
Palmer, 1989	1	1	0	0	1	0	1	1	5
Molina, 1989	1	1	0	1	1	0	1	0	5
Vall Mayans, 1990	1	1	0	1	1	0	1	0	5
Kew, 1990	1	0	0	1	1	0	1	1	5
Yu, 1991	1	1	1	0	0	0	1	0	4
Hsing, 1992	1	0	0	1	2	0	1	0	5
Khella, 1992	0	1	0	0	1	0	1	1	4
Braga, 1997	1	1	0	1	2	0	1	1	7
Heinemann, 1997	1	1	0	1	1	0	1	0	5
Yu, 2003	1	1	0	1	1	0	1	1	6
Kanazir, 2010	0	1	0	0	0	0	1	1	3
Hassan, 2017	1	1	0	1	2	0	1	1	7

[a] Defined as adjustment for at least age, smoking, alcohol intake, and a measure of socioeconomic status.

Supplementary Table 5. Newcastle-Ottawa Quality grading of prospective cohort studies included in systematic review and meta-analysis.

First author, publication year (cohort)	Quality measure									**Total quality score (out of 9)**
	Representative study	Selection of non-exposed cohort	Ascertainment of exposure (oral contraceptive use)	Outcome not present at baseline	Comparable exposure	Appropriate adjustment [a]	Record linkage used for outcome	Follow-up, >4 years	Adequacy of follow-up	
Rosenblatt, 2009 (FTW)	0	1	1	0	1	0	1	1	1	6
Vessey, 2013 (Ox-FPA)	1	1	1	1	1	0	1	1	1	8
McGlynn, 2015 (LCPP)	1	1	1	1	1	1	1	1	1	9
Iversen, 2017 (RCGP)	1	1	1	1	1	0	1	1	1	8
Petrick, 2020 (LCPP)	1	1	1	1	1	1	1	1	1	9
Tuo, 2022 (SWHS)	1	1	1	1	1	1	1	1	1	9
Current analysis (MWS)	1	1	1	1	1	1	1	1	1	9
Current analysis (UK Biobank)	1	1	1	1	1	1	1	1	1	9

[a] Defined as adjustment for at least age, smoking, alcohol intake, and a measure of socioeconomic status.

Abbreviations: FTW, Shanghai Female Textile Workers; LCPP, Liver Cancer Pooling Project; MWS, Million Women Study; Ox-FPA, Oxford-Family Planning Association; RCGP, Royal College of General Practitioners' Oral Contraception Study; SWHS, Shanghai Women's Health Study.

Supplementary Figures

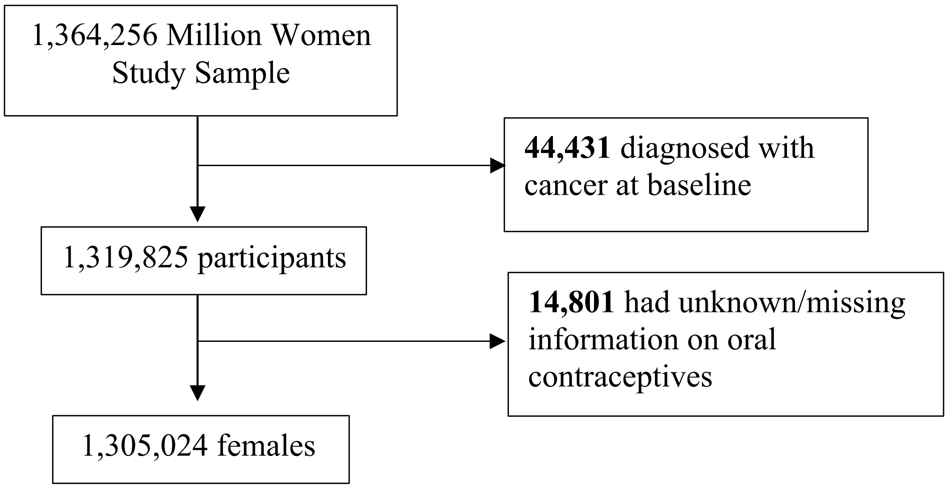

Supplementary Figure 1. Flow chart of exclusion criteria applied to the Million Women Study sample.

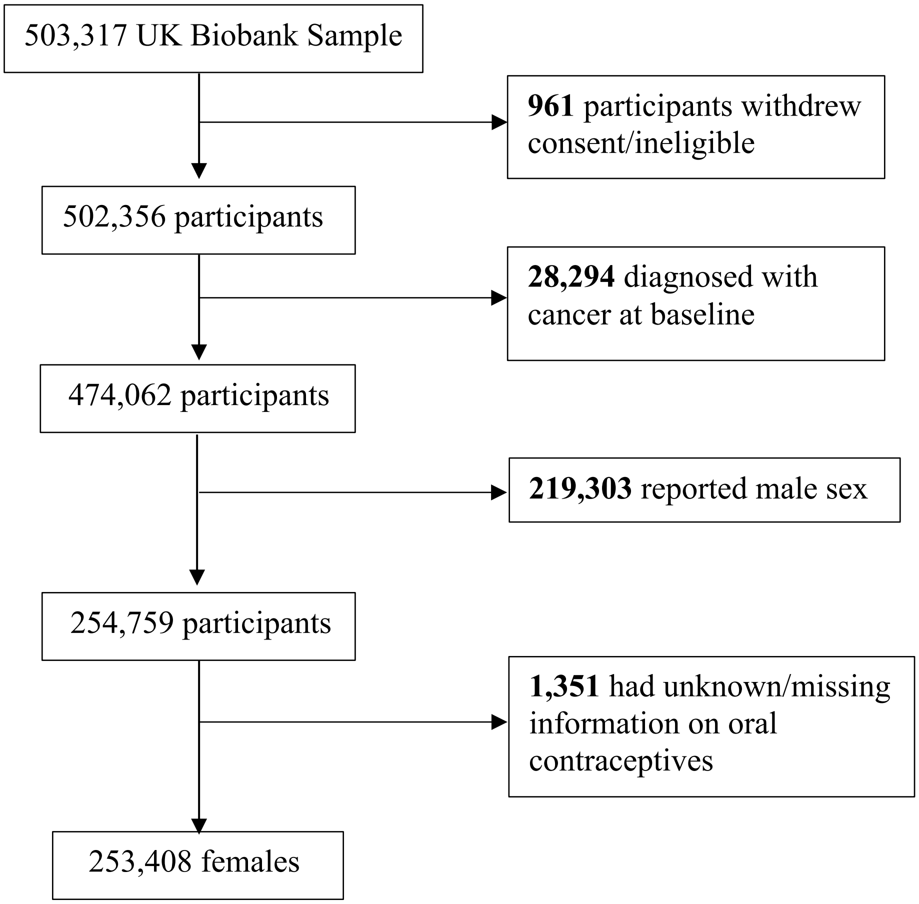

Supplementary Figure 2. Flow chart of exclusion criteria applied to the UK Biobank sample.

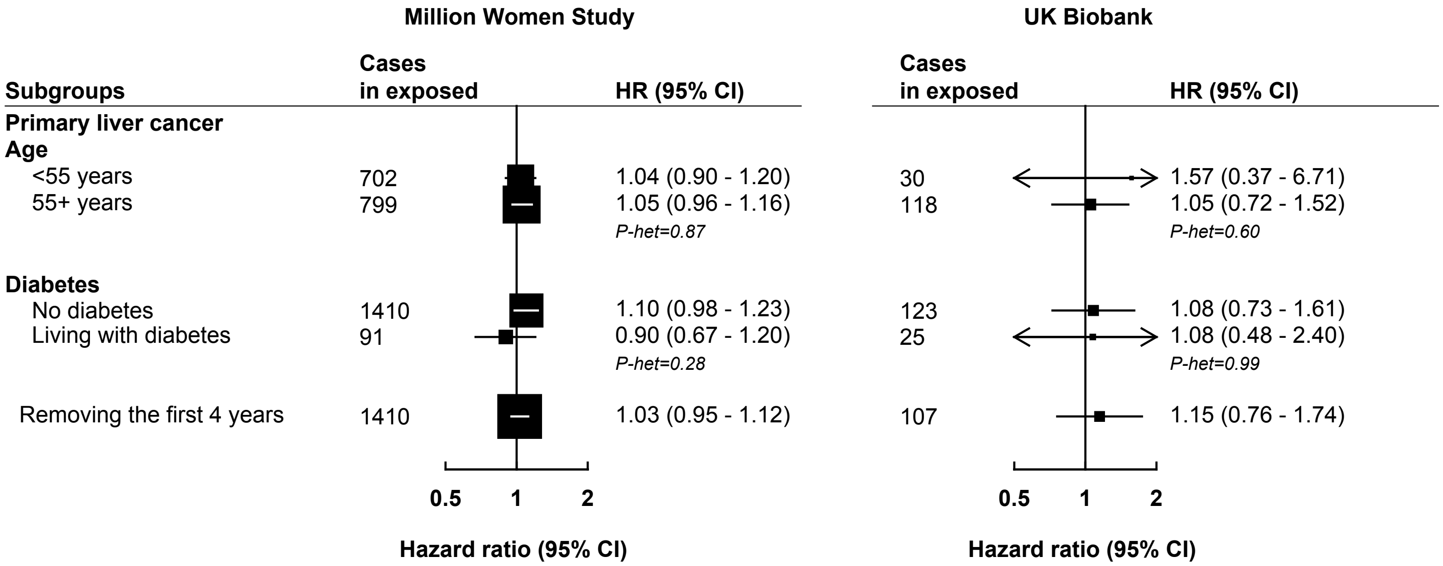

Supplementary Figure 3. Subgroup and sensitivity analyses for ever versus never oral contraceptive use and risk of liver cancer in Million Women Study and UK Biobank.

Never use of oral contraceptives was the reference group.

Cases in the exposed represents the total number of cases who had ever used oral contraceptives.

Million Women Study analyses were stratified by year of recruitment, year of birth, and adjusted for deprivation (Townsend deprivation index quintiles), region of recruitment, body mass index, height, physical activity, smoking status, alcohol consumption, education, diabetes status, menopausal status/years since menopause, menopausal hormone therapy use, and history of a hysterectomy.

UK Biobank analyses were stratified by age at recruitment, and adjusted for region of recruitment, deprivation, body mass index, height, physical activity, smoking status, alcohol consumption, coffee intake, ethnicity, education, diabetes status, menopausal status/years since menopause, menopausal hormone therapy use, history of a hysterectomy.

P-het represents the p-value for heterogeneity between subgroups obtained from χ^2 for the difference between hazard ratios of liver cancer associated with ever versus never use of oral contraceptives for the subgroups of interest.

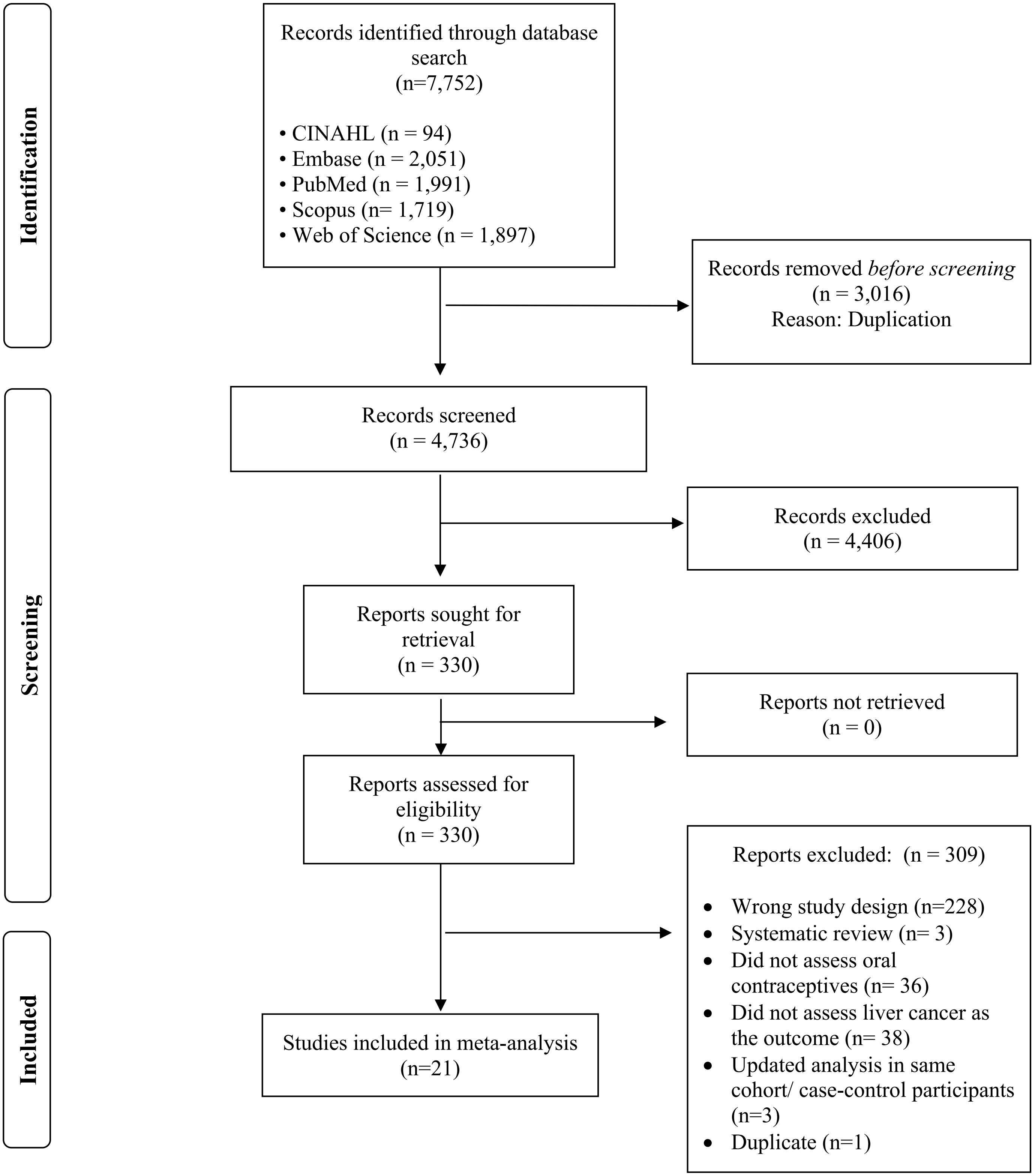

Supplementary Figure 4. PRISMA flow diagram of the systematic review literature search of observational studies assessing oral contractive use in relation to liver cancer risk.

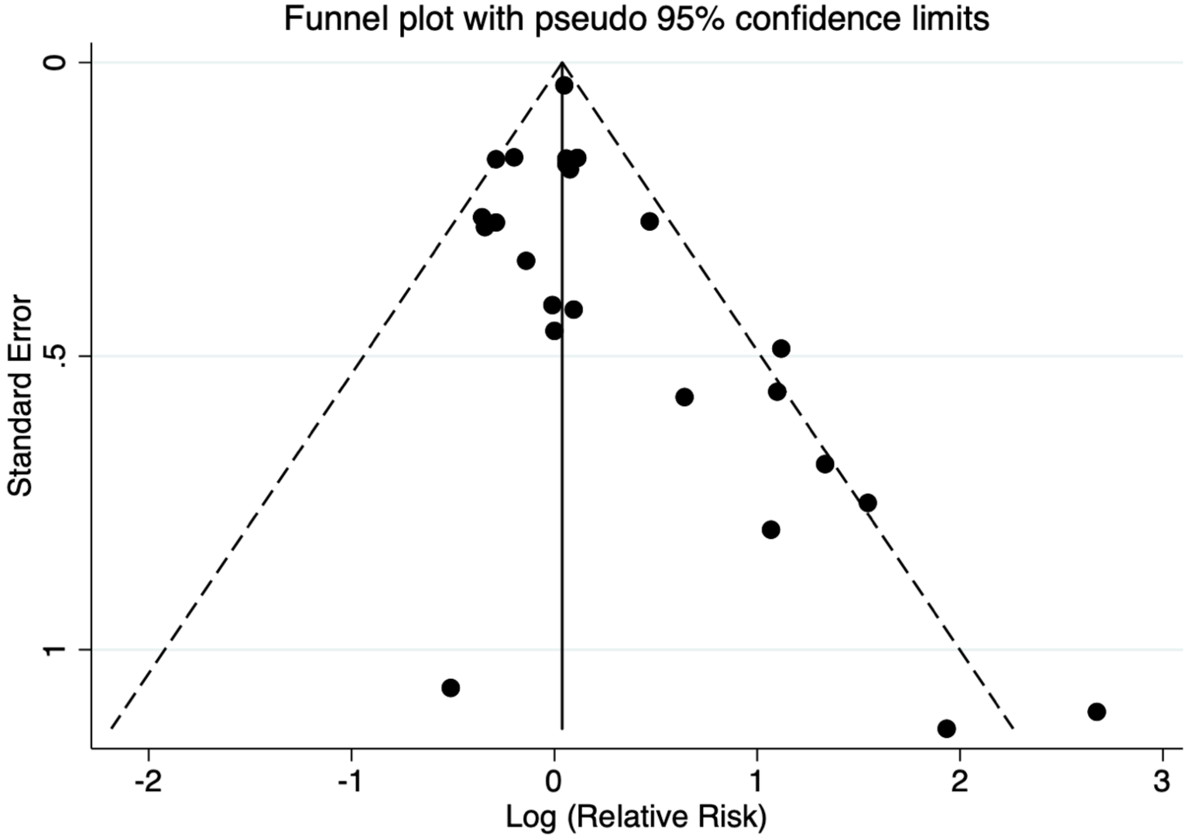

Supplementary Figure 5. Funnel plot with pseudo 95% confidence intervals for ever versus never use of oral contraceptives and liver cancer risk.

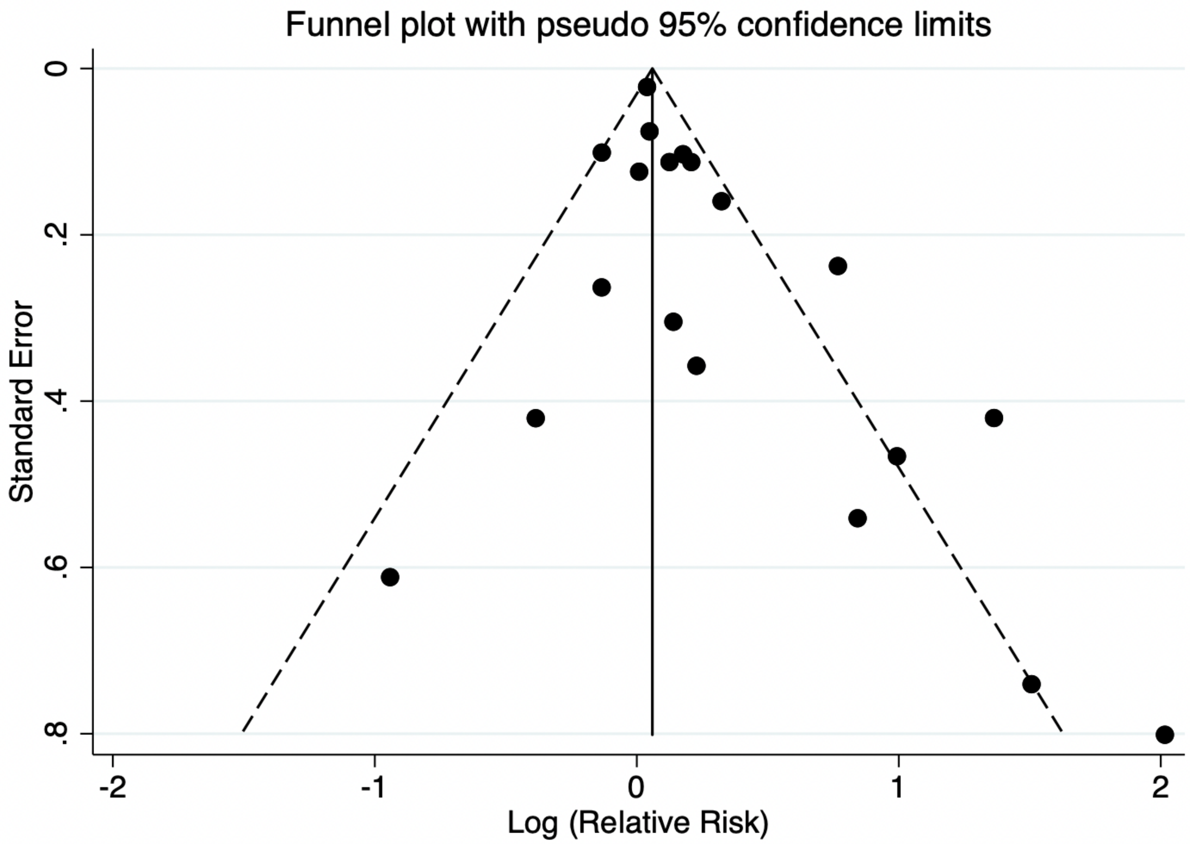

Supplementary Figure 6. Funnel plot with pseudo 95% confidence intervals for long duration of oral contraceptives (per 5 years of use) and liver cancer risk.

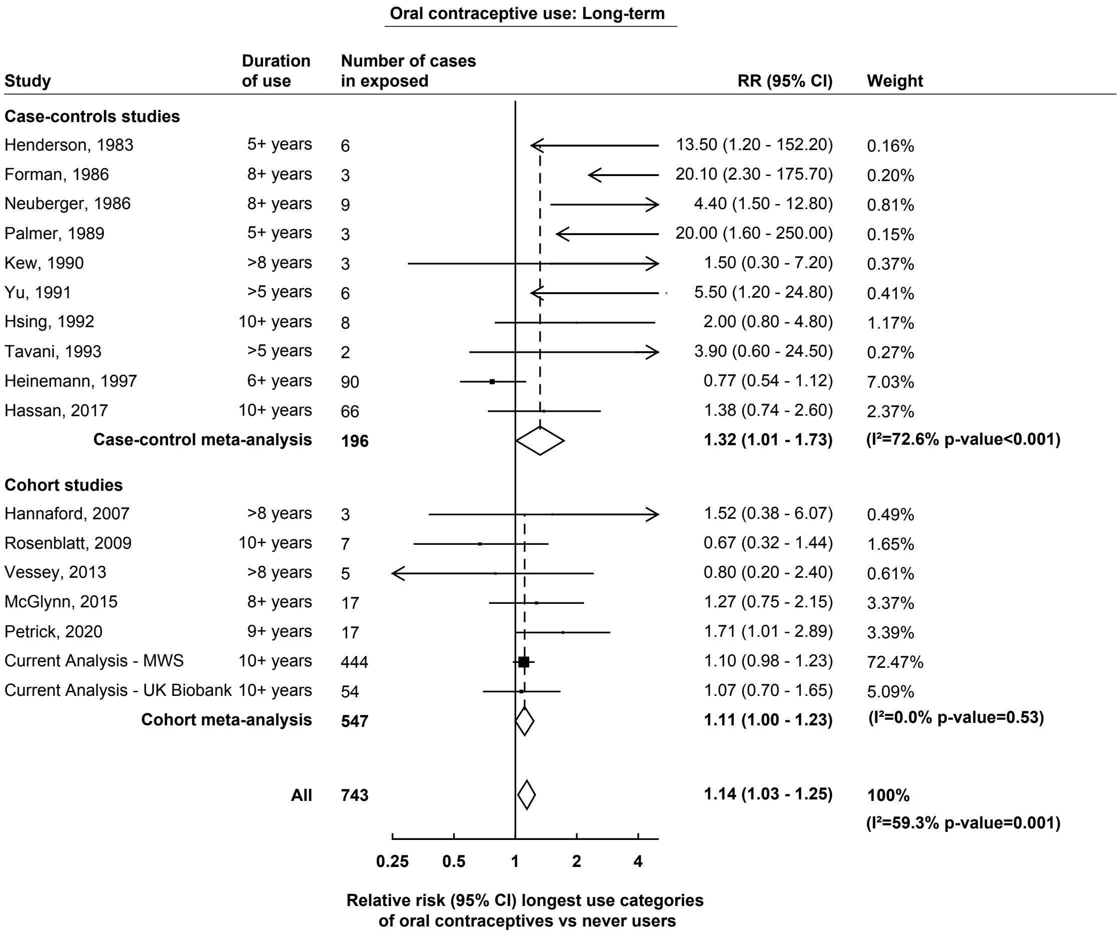

Supplementary Figure 7. Meta-analysis of highest duration of oral contraceptive use categories compared to never users and risk of liver cancer

Meta-analysis estimates are derived from fixed effects models.

Abbreviations: CI, confidence intervals; MWS, Million Women Study; RR, relative risk.

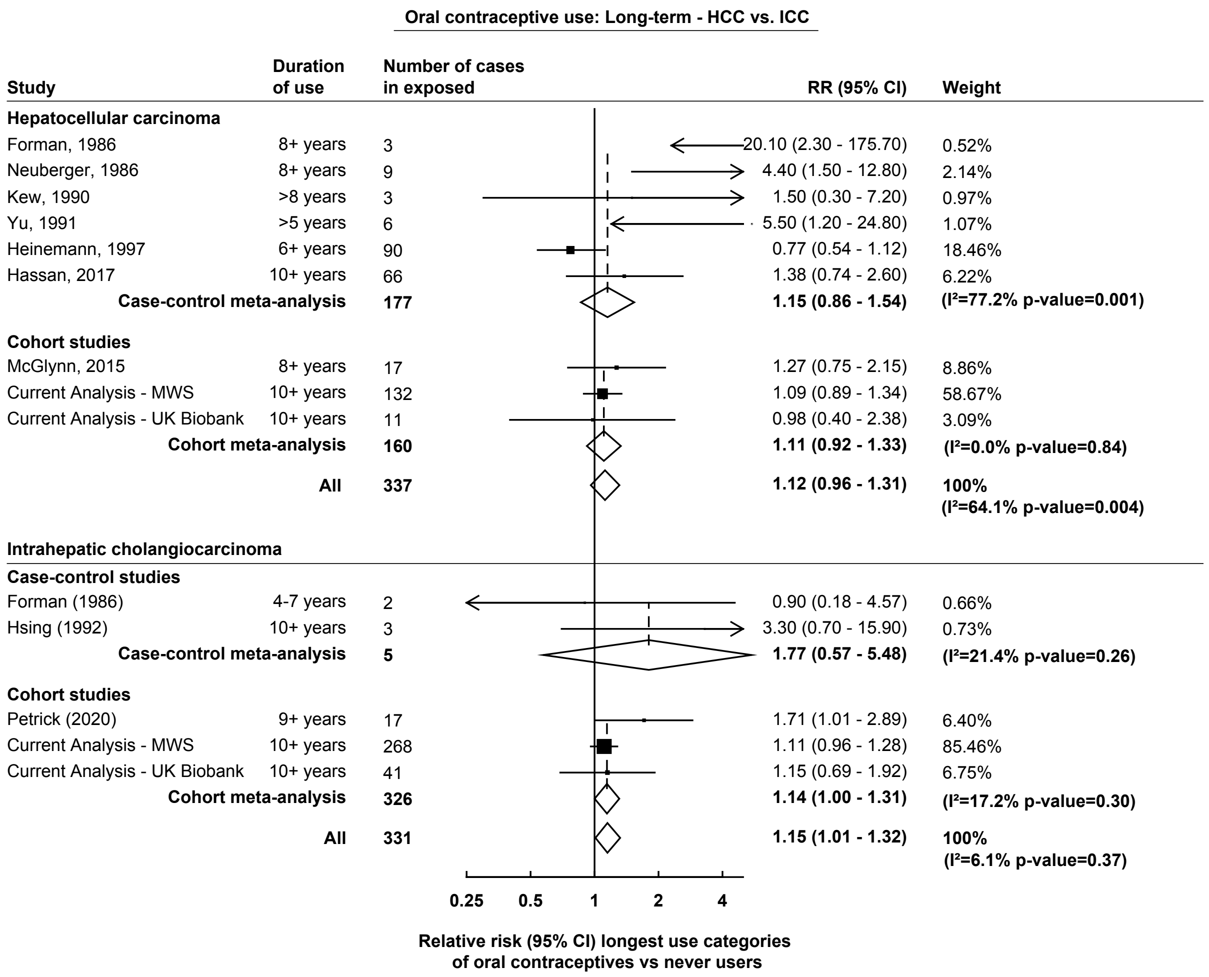

Supplementary Figure 8. Meta-analysis of highest duration of oral contraceptive use categories compared to never users and risk of hepatocellular carcinoma and intrahepatic cholangiocarcinoma.

Meta-analysis estimates are derived from fixed effects models.

Abbreviations: CI, confidence intervals; HCC, hepatocellular carcinoma; ICC, intrahepatic cholangiocarcinoma; MWS, Million Women Study; RR, relative risk.

PROSPERO protocol

NIHR | National Institute for Health and Care Research — PROSPERO International prospective register of systematic reviews

Exogenous hormone use and risk of liver cancer

Katherine McGlynn, Cody Watling, Aika Wojt, Gisela Butera

To enable PROSPERO to focus on COVID-19 submissions, this registration record has undergone basic automated checks for eligibility and is published exactly as submitted. PROSPERO has never provided peer review, and usual checking by the PROSPERO team does not endorse content. Therefore, automatically published records should be treated as any other PROSPERO registration. Further detail is provided here.

Citation
Katherine McGlynn, Cody Watling, Aika Wojt, Gisela Butera. Exogenous hormone use and risk of liver cancer. PROSPERO 2024 Available from https://www.crd.york.ac.uk/PROSPERO/view/CRD42024552518

REVIEW TITLE AND BASIC DETAILS

Review title
Exogenous hormone use and risk of liver cancer

Review objectives
Primary question(s): (1) Does the use of oral contraceptives influence liver cancer risk? (2) Does the use of menopausal hormone therapy influence liver cancer risk?

Keywords
Exogenous hormones, hepatocellular carcinoma, hormone replacement therapy, menopausal hormone therapy, oestrogen-progesterone, oral contraceptives

SEARCHING AND SCREENING

Searches
Electronic databases to be searched include: EMBASE (Elsevier), PubMed/MEDLINE (NLM), Scopus (Elsevier), and Web of Science: Core Collection (Clarivate Analytics)
Studies will not be excluded if published in a language other than English and there is no reliable translation is available. Additionally, we will not limit our literature review to certain publication years.
Case reports, abstract-only publications, commentaries, editorials, and other non-peer reviewed publications will be excluded from the systematic review.

Study design
Inclusion criteria: We will include all observational studies that assessed oral contraceptive or menopausal hormone therapy use and risk of liver cancer
Exclusion criteria: non-human studies, case reports, abstract-only publications, commentaries, editorials, and other non-peer reviewed publications will be excluded from the systematic review.

ELIGIBILITY CRITERIA

Condition or domain being studied
Liver cancer is the third most common cause of cancer death globally. Primary liver cancer can be subdivided into two subtypes; hepatocellular carcinoma accounts for 75-85% of all cases of primary liver cancer whereas intrahepatic cholangiocarcinoma, accounts for 10-20% of liver cancer cases.

Population
Inclusion: Human female adults (18+ years old) - all observational studies conducted worldwide.
Exclusion: Cell studies, animal models.

Intervention(s) or exposure(s)
Primary exposure: Oral contraceptive use and menopausal hormone therapy use; participants will be categorised as never/former/current or never/ever. We will also look at duration of use and formulation types of oral contraceptive and menopausal hormone therapy.

Comparator(s) or control(s)
Not applicable; participants

OUTCOMES TO BE ANALYSED

Main outcomes
Incidence of liver cancer. We will also look at hepatocellular carcinoma and intrahepatic cholangiocarcinoma separately.

Measures of effect
Relative risks, Odds Ratios, Hazard Ratios.

Additional outcomes
Not applicable.

DATA COLLECTION PROCESS

Data extraction (selection and coding)
Measures of association, author, year of publication, setting and study design, study location, exposure and outcome measurements, and results will be extracted.
Step 1. Protocol and Database Search – The research team will develop a protocol with a medical librarian. Then, Covidence screening software will be used to screen references that result from the literature search; additional duplicates will be removed. Prior to the beginning title and abstract screening stage, two authors will pilot the protocol in Covidence to ensure consistency in screening between reviewers.
Step 2. Title and Abstract Screening – Titles and abstracts resulting from the literature search will be screened for inclusion by two separate reviewers (CW and AW). If disagreement exists between the two primary reviewers, a third reviewer (KAM) will determine the final inclusion decision.
Step 3. Full-text screening – All literature included in Step 2 will be reviewed based on the full text by both reviewers (CW and AW). If disagreement exists, a third reviewer (KAM) will determine final inclusion.
Step 4. Extraction – Data extraction will be completed with Covidence, and Excel, if necessary, from studies selected from Step 3. A data extraction form will be developed and first piloted by two reviewers (CW and AW), who will meet to review and resolve any inconsistencies in the data extraction through discussion and consensus. After piloting the data extraction form, the primary investigator (CW) and the other reviewer (AW) will extract all necessary data; study team members will flag any discrepancies, and review and if any disagreement persists, KAM will determine the final conclusion.

Risk of bias (quality) assessment
Methodological quality will be assessed by the Newcastle-Ottawa assessment scale. The primary investigator (CW) will grade the certainty of the evidence alongside another researcher (AW).

PLANNED DATA SYNTHESIS

Strategy for data synthesis
Data will be combined statistically in Stata statistical software. We will compute pooled measures of association (e.g., risk ratios and 95% confidence intervals) for associations between 1) oral contraceptive use and liver cancer risk (never vs. ever/ never, former, and current) and 2) menopausal hormone therapy use (never vs. ever/ never, former, and current). We will also look at meta-analysing results by duration, if available.
Potential sources of effect heterogeneity will be assessed via two statistical tests: the χ^2 test and the I^2 test. Sensitivity analyses of liver cancer subtype, study design, and study population will also be examined as potential sources of bias if sufficient amount of data exists.

Analysis of subgroups or subsets
Liver cancer subtypes (e.g., hepatocellular carcinoma (HCC) and/or intrahepatic cholangiocarcinoma (ICC)) will assessed in additional analyses if there are sufficient number of cases.

REVIEW AFFILIATION, FUNDING AND PEER REVIEW

Review team members

- Dr Katherine McGlynn, National Cancer Institute
- Dr Cody Watling, National Cancer Institute
- Ms Aika Wojt, National Cancer Institute
- Ms Gisela Butera, National Cancer Institute

Review affiliation
National Cancer Institute

Funding source
NIH Intramural

Named contact
Katherine McGlynn. National Cancer Institute, NIH 9609 Medical Center Drive, Rm 6E-446 Rockville, MD 20850
mcglynnk@mail.nih.gov

TIMELINE OF THE REVIEW

Review timeline
Start date: 01 June 2024. End date: 31 December 2024

Date of first submission to PROSPERO
11 June 2024

Date of registration in PROSPERO
22 June 2024

CURRENT REVIEW STAGE

Publication of review results
The intention is to publish the review once completed.The review will be published in English

Stage of the review at this submission

Review stage	Started	Completed
Pilot work		
Formal searching/study identification		
Screening search results against inclusion criteria		
Data extraction or receipt of IP		
Risk of bias/quality assessment		
Data synthesis		

Review status
The review is currently planned or ongoing.

ADDITIONAL INFORMATION

PROSPERO version history

- Version 1.1 published on 22 Jun 2024
- Version 1.0 published on 22 Jun 2024

Review conflict of interest
None known

Country
United States of America

Medical Subject Headings
Contraceptives, Oral; Female; Hormones; Humans; Liver Neoplasms; Menopause

Disclaimer
The content of this record displays the information provided by the review team. PROSPERO does not peer review registration records or endorse their content.

PROSPERO accepts and posts the information provided in good faith; responsibility for record content rests with the review team. The owner of this record has affirmed that the information provided is truthful and that they understand that deliberate provision of inaccurate information may be construed as scientific misconduct.

PROSPERO does not accept any liability for the content provided in this record or for its use. Readers use the information provided in this record at their own risk.

Any enquiries about the record should be referred to the named review contact

PRISMA 2020 Checklist

Section and Topic	Item #	Checklist item	Location where item is reported
**TITLE**			
Title	1	Identify the report as a systematic review.	Title
**ABSTRACT**			
Abstract	2	See the PRISMA 2020 for Abstracts checklist.	Complete
**INTRODUCTION**			
Rationale	3	Describe the rationale for the review in the context of existing knowledge.	Page 2
Objectives	4	Provide an explicit statement of the objective(s) or question(s) the review addresses.	Page 2
**METHODS**			
Eligibility criteria	5	Specify the inclusion and exclusion criteria for the review and how studies were grouped for the syntheses.	Methods/ Appendix
Information sources	6	Specify all databases, registers, websites, organisations, reference lists and other sources searched or consulted to identify studies. Specify the date when each source was last searched or consulted.	Methods/ Appendix
Search strategy	7	Present the full search strategies for all databases, registers and websites, including any filters and limits used.	Appendix
Selection process	8	Specify the methods used to decide whether a study met the inclusion criteria of the review, including how many reviewers screened each record and each report retrieved, whether they worked independently, and if applicable, details of automation tools used in the process.	Appendix
Data collection process	9	Specify the methods used to collect data from reports, including how many reviewers collected data from each report, whether they worked independently, any processes for obtaining or confirming data from study investigators, and if applicable, details of automation tools used in the process.	Appendix
Data items	10a	List and define all outcomes for which data were sought. Specify whether all results that were compatible with each outcome domain in each study were sought (e.g. for all measures, time points, analyses), and if not, the methods used to decide which results to collect.	Appendix
	10b	List and define all other variables for which data were sought (e.g. participant and intervention characteristics, funding sources). Describe any assumptions made about any missing or unclear information.	Appendix
Study risk of bias assessment	11	Specify the methods used to assess risk of bias in the included studies, including details of the tool(s) used, how many reviewers assessed each study and whether they worked independently, and if applicable, details of automation tools used in the process.	Page 4
Effect measures	12	Specify for each outcome the effect measure(s) (e.g. risk ratio, mean difference) used in the synthesis or presentation of results.	Page 4
Synthesis methods	13a	Describe the processes used to decide which studies were eligible for each synthesis (e.g. tabulating the study intervention characteristics and comparing against the planned groups for each synthesis (item #5)).	Appendix
	13b	Describe any methods required to prepare the data for presentation or synthesis, such as handling of missing summary statistics, or data conversions.	Appendix
	13c	Describe any methods used to tabulate or visually display results of individual studies and syntheses.	Appendix
	13d	Describe any methods used to synthesize results and provide a rationale for the choice(s). If meta-analysis was performed, describe the model(s), method(s) to identify the presence and extent of statistical heterogeneity, and software package(s) used.	Page 4
	13e	Describe any methods used to explore possible causes of heterogeneity among study results (e.g. subgroup analysis, meta-regression).	Appendix
	13f	Describe any sensitivity analyses conducted to assess robustness of the synthesized results.	Appendix

PRISMA 2020 Checklist

Section and Topic	Item #	Checklist item	Location where item is reported
Reporting bias assessment	14	Describe any methods used to assess risk of bias due to missing results in a synthesis (arising from reporting biases).	Appendix
Certainty assessment	15	Describe any methods used to assess certainty (or confidence) in the body of evidence for an outcome.	Page 4/ Appendix
**RESULTS**			
Study selection	16a	Describe the results of the search and selection process, from the number of records identified in the search to the number of studies included in the review, ideally using a flow diagram.	Page 7/8
	16b	Cite studies that might appear to meet the inclusion criteria, but which were excluded, and explain why they were excluded.	Appendix
Study characteristics	17	Cite each included study and present its characteristics.	Appendix
Risk of bias in studies	18	Present assessments of risk of bias for each included study.	Appendix
Results of individual studies	19	For all outcomes, present, for each study: (a) summary statistics for each group (where appropriate) and (b) an effect estimate and its precision (e.g. confidence/credible interval), ideally using structured tables or plots.	Page 7/8
Results of syntheses	20a	For each synthesis, briefly summarise the characteristics and risk of bias among contributing studies.	Page 8
	20b	Present results of all statistical syntheses conducted. If meta-analysis was done, present for each the summary estimate and its precision (e.g. confidence/credible interval) and measures of statistical heterogeneity. If comparing groups, describe the direction of the effect.	Page 8
	20c	Present results of all investigations of possible causes of heterogeneity among study results.	Page 8
	20d	Present results of all sensitivity analyses conducted to assess the robustness of the synthesized results.	Appendix
Reporting biases	21	Present assessments of risk of bias due to missing results (arising from reporting biases) for each synthesis assessed.	Appendix
Certainty of evidence	22	Present assessments of certainty (or confidence) in the body of evidence for each outcome assessed.	Appendix
**DISCUSSION**			
Discussion	23a	Provide a general interpretation of the results in the context of other evidence.	Page 9-11
	23b	Discuss any limitations of the evidence included in the review.	Page 11
	23c	Discuss any limitations of the review processes used.	Page 11
	23d	Discuss implications of the results for practice, policy, and future research.	Page 9-11
**OTHER INFORMATION**			
Registration and protocol	24a	Provide registration information for the review, including register name and registration number, or state that the review was not registered.	Page 5
	24b	Indicate where the review protocol can be accessed, or state that a protocol was not prepared.	Page 5
	24c	Describe and explain any amendments to information provided at registration or in the protocol.	N/A
Support	25	Describe sources of financial or non-financial support for the review, and the role of the funders or sponsors in the review.	Page 5
Competing interests	26	Declare any competing interests of review authors.	Page 11

PRISMA 2020 Checklist

Section and Topic	Item #	Checklist item	Location where item is reported
Availability of data, code and other materials	27	Report which of the following are publicly available and where they can be found: template data collection forms; data extracted from included studies; data used for all analyses; analytic code; any other materials used in the review.	Page 11

From: Page MJ, McKenzie JE, Bossuyt PM, Boutron I, Hoffmann TC, Mulrow CD, et al. The PRISMA 2020 statement: an updated guideline for reporting systematic reviews. BMJ 2021;372:n71. doi: 10.1136/bmj.n71
